# Osimertinib versus platinum-pemetrexed in patients with previously treated EGFR T790M advanced non-small cell lung cancer: An updated AURA3 trial-based cost-effectiveness analysis

**DOI:** 10.3389/fonc.2022.833773

**Published:** 2022-10-17

**Authors:** Yin Shi, Rui Pei, Shao Liu

**Affiliations:** ^1^ Department of Pharmacy, Xiangya Hospital, Central South University, Changsha, China; ^2^ National Clinical Research Center for Geriatric Disorders, Xiangya Hospital, Central South University, Changsha, China; ^3^ The Hunan Institute of Pharmacy Practice and Clinical Research, Changsha, China

**Keywords:** osimertinib, non-small-cell lung cancer, EGFR, cost-effectiveness, platinum-pemetrexed

## Abstract

**Background:**

A recently overall survival (OS) analysis from the AURA3 trial indicated that osimertinib improves median OS versus platinum-pemetrexed for patients with previously treated epidermal growth factor receptor (EGFR) T790M advanced non-small cell lung cancer (NSCLC). Here, we assessed the cost-effectiveness of second-line osimertinib versus platinum-pemetrexed, from the perspectives of the United States payer and the Chinese health care system.

**Methods:**

A Markov model was constructed to compare the costs and health outcomes of osimertinib versus platinum-pemetrexed in second-line treatment of EGFR T790M advanced NSCLC. Life years (LYs), quality adjusted life years (QALYs), costs, and incremental cost-effectiveness ratios (ICERs) were calculated. One-way and probabilistic sensitivity analyses assessed the robustness of the model. Cost-effectiveness was examined in the intention-to-treat (ITT) population and central nervous system (CNS) metastases population.

**Results:**

In the United States, compared with platinum-pemetrexed, osimertinib yielded additional effectiveness of 0.43 QALYs and -0.12 QALYs, with incremental costs of $67,588 and $16,465 in the ITT population and CNS metastases population, respectively. The ICERs of osimertinib over platinum-pemetrexed were $159,126/QALY and $-130,830/QALY, respectively. The probability of osimertinib being cost-effective was 37% and 5.76%, respectively, at the willingness-to-pay (WTP) threshold of $150,000/QALY. In China, osimertinib showed incremental effectiveness of 0.34 QALYs and -0.14 QALYs, with incremental costs of $1,663 and $-505, resulting in ICERs of $4,950/QALY and $3,754/QALY in the ITT population and CNS metastases population, respectively. At the WTP threshold of $37,489/QALY, there was a 100% and 26% likelihood that osimertinib was cost-effective in the ITT population and CNS metastases population.

**Conclusion:**

In the United States, second-line osimertinib treatment for EGFR T790M advanced NSCLC is not cost-effective compared to platinum-pemetrexed under the current WTP threshold. When the osimertinib price reduces, the economic outcome may become favorable. In China, assuming a WTP threshold of $37,489/QALY, osimertinib is the dominant treatment strategy compared with platinum-pemetrexed in the ITT population and provides cost savings for CNS metastases patients.

## Introduction

Lung cancer remains the primary cause of tumor-related deaths (1). In the United States, there are expected to be 235,760 new cases of lung and bronchus cancer and 131,880 related deaths in 2021 (2). In China, 820,000 new cases and 720,000 deaths were reported in 2020 (3). Non-small cell lung cancer (NSCLC) accounts for approximately 85% of all lung cancer subtypes (4). More than 30% of patients with NSCLC are initially diagnosed as advanced diseases and are unresectable (5). Most patients develop disease progression after chemoradiotherapy, and the five-year survival rate is only 15%-30% (6). First-line treatment with first- or second-generation epidermal growth factor receptor (EGFR) tyrosine kinase inhibitors (TKIs) significantly prolongs survival compared with chemotherapy (7). However, after nine-13 months of first- or second-generation TKI treatment, drug resistance is often inevitable, and the EGFR T790M mutation is the main cause of drug resistance (8).

Osimertinib is a selective third-generation EGFR-TKI that irreversibly inhibits EGFR and EGFR T790M; its activity in patients with central nervous system (CNS) metastases is superior to that of first- and second-generation EGFR-TKIs (9). A phase III study, AURA3, showed that for patients with EGFR T790M advanced NSCLC previously treated with EGFR-TKI, the progression-free survival (PFS) of osimertinib arm was 10.1 months, significantly higher than that of the platinum-pemetrexed arm (4.4 months) (10). Additionally, the incidence of grade 3 or above treatment-related adverse events (AEs) in the osimertinib arm was much lower than that in the platinum-pemetrexed arm (9% versus 34%). Based on the mature overall survival (OS) analysis from the AURA3 trial, osimertinib improved survival at 24 and 36 months versus chemotherapy (55% versus 43% and 37% versus 30%, respectively), with a longer median OS (26.8 months versus 22.5 months, respectively) (11). osimertinib in second-line treatment of EGFR T790M positive NSCLC after progression of first- or second-generation EGFR-TKI therapy has been included in the National Comprehensive Cancer Network guidelines (12).

In recent years, the burden of cancer has increased in both developed and developing countries (5). Despite the significant health outcomes of second-line osimertinib treatment, there is growing concern about its high financial burden on patients and society. Thus, we evaluated the cost-effectiveness of osimertinib versus chemotherapy for previously-treated EGFR T790M positive NSCLC from the perspectives of the United States payer and Chinese healthcare system, based on updated data from the AURA3 trial.

## Materials and methods

### Patients and intervention

We constructed a cost-effectiveness model to estimate the cost inputs and effectiveness of osimertinib and platinum-pemetrexed second-line treatment in patients with EGFR T790M advanced NSCLC. The cohort we modeled was based on the AURA3 trial and its updated OS analysis (10, 11). Individuals had a median age of 62 years and were randomly assigned to receive platinum-pemetrexed chemotherapy or osimertinib. Details of patient characteristics are provided in **Supplementary Table 1**.

### Model structure

**Figure 1:**
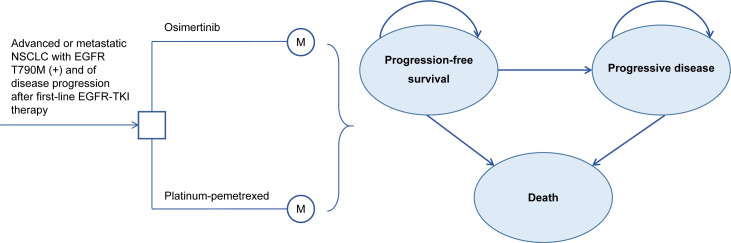
Three-state Markov model Structure. NSCLC, Non-Small Cell Lung Cancer; EGFR, epidermal growth factor receptor; TKI, tyrosine kinase inhibitor.

Our study followed the Consolidated Health Economic Evaluation Reporting Standards (CHEERS) reporting guideline for economic evaluations (13) (**Supplementary Table 6**). This model-based study used published trial data with no human participants involved and does not require institutional review board approval by an ethics committee. A three health states Markov model was established by TreeAge Pro 2021 (TreeAge Software, Williamstown, MA, USA) (**Figure 1**). We assumed that the model cycle length was three weeks, which was consistent with the administration schedules in the AURA3 trial (10). The time horizon is the lifetime. All populations were in PFS state when entering the model and were assumed to receive osimertinib (oral, 80 mg) once a day or four-cycle cisplatin plus pemetrexed chemotherapy (75 mg/m^2^ cisplatin and 500 mg/m^2^ pemetrexed, both intravenously) every three weeks followed by pemetrexed maintenance therapy until progression of a disease or unacceptable AEs. Upon progression, 53% and 81% of patients in the osimertinib and platinum-pemetrexed groups received subsequent therapy and best supportive care (BSC) until death (11). The option of subsequent treatment was based on the information from the AURA3 OS analysis (11) and is shown in **Supplementary Table 2**. We also considered the AE-related treatment discontinuation of patients, which was 7% in the osimertinib group and 10% in the platinum-pemetrexed group (10). Life years (LYs), quality adjusted life years (QALYs), total costs, and incremental cost-effectiveness ratios (ICERs) were measured. QALYs can comprehensively reflect the patient’s length and quality of life, and ICER is the ratio of cost difference and QALY difference between two treatment strategies, which is used to evaluate the cost-effectiveness between treatment strategies. The willingness-to-pay (WTP) represents the highest price decision makers are willing to sacrifice for health gains and its threshold in China and the United States was $150,000/QALY and $37,489/QALY (three times the per capita gross domestic product of China in 2021), respectively (14–16). This study applied half-cycle correction and an annual discount rate of 3% and 5% in costs as well as health utilities in the United States and Chinese contexts, respectively (17, 18).

### Model transition probabilities

The probability of progression and death for both osimertinib and platinum-pemetrexed groups were derived from the PFS and OS Kaplan-Meier survival curves in the AURA3 trial (10, 11). We used the GetData Graph Digitizer 2.26 to extract the data points of published Kaplan-Meier survival curves. We used the method of Hoyle et al. (Website for statistical code: https://www.mq.edu.au/research/research-centres-groups-and-facilities/prosperous-economies/centres/centre-for-the-health-economy/our-people/team-bios/prof-hoyle.) to recreate individual patient-level data and fitted the data into the most common parametric survival functions (19). As only the “R” statistics code for fitting the survival curves to the Weibull survival function is provided by Hoyle et al. (19). this study has made the necessary additions to the statistical codes for fitting other common survival functions (exponential, log-logistic, and lognormal) (**Supplementary Table 4**). We choose the most appropriate fit parametric distribution based on Akaike information and Bayesian information criteria, as well as clinical practice (**Supplementary Table 5**). For the ITT population, the Weibull distribution was the best fit distribution for PFS and OS curves of the platinum-pemetrexed group, and the PFS and OS curves of the osimertinib group were fitted using a log-logistic distribution (**Supplementary Figures 1**, **2**). For patients with CNS metastases, the log-logistic distribution and Weibull distribution provided the best fit for PFS Kaplan-Meier survival curves of the osimertinib and platinum-pemetrexed groups, respectively (**Supplementary Figure 3**). The detailed parameters of the survival functions are outlined in **Supplementary Table 2**. Due to a lack of OS Kaplan-Meier survival curves of patients with CNS metastases, we assumed that the OS rate of the platinum-pemetrexed group in patients with CNS metastases was consistent with that of the platinum-pemetrexed group in the ITT population. For patients with CNS metastases, the OS rate in the osimertinib arm was calculated by multiplying the HRs of OS of osimertinib versus platinum-pemetrexed and the OS rate in the platinum-pemetrexed group of patients with CNS metastases.

### Costs

Direct medical costs including drug acquisition, radiotherapy, chemotherapy intravenous infusion, EGFR mutation testing, follow-up, BSC, end-of-life, and AE management costs were covered in our analysis (**Supplementary Table 3**). In the United States, drug acquisition costs were based on the Centers for Medicare & Medicaid Services’ average sale price of 2021 and Drug.com, a website that provides accurate and independent data sources based on IBM Watson Micromedex, Cerner Multum™ American Society of Hospital Pharmacists, and others (20, 21). Radiotherapy and chemotherapy infusion costs were acquired from the 2021 Medicare physician fee schedule and Medicare fee-for-service payment (22, 23). EGFR mutation testing, follow-up, BSC, end-of-life, and AE management costs were estimated based on published literature (24–28). In China, the costs of drug acquisition were derived from the payment standards of the List of Medicines Insured in 2020 by the National Healthcare Security Administration and the purchase price of medical institutions published on drug procurement platforms (29, 30). The EGFR mutation testing cost was based on the current local charge and the rest of the costs were obtained from other published cost-effectiveness analyses (31–36). All costs were shown in US dollars (1 US dollar = 6.48 Chinese yuan) and inflated to 2021 US dollars using the Consumer Price Index (37, 38).

We assumed that the average body surface area (BSA) were 1.79 m^2^ and 1.72 m^2^ and the bodyweight of 70 kg and 65 kg in American and Chinese cohorts, respectively, to calculate dose administration (39–41). AEs (grade ≥3) with a greater than 5% difference in incidence rate between the osimertinib and platinum-pemetrexed groups in the AURA3 trial (11) were incorporated into the model. The cost of end-of-life care was considered a one-off cost and assumed to be the same in both treatment strategies.

### Utilities

Health utilities are one of the primary outcomes used to calculate QALYs in cost-effectiveness analyses and have significant differences among different countries (42). QALYs were estimated by weighting patients’ accumulated LYs based on the utility value of the corresponding health state, which can comprehensively reflect the patient’s length and quality of life. In the United States context, health utilities were derived from the literature that obtained the health utility scores of metastatic NSCLC in a North American setting by assessing the EuroQol five-dimension scale and transforming it (43). Utility values in the context of Chinese were derived from a study by Yunjie et al. (42), which assessed the health utilities of advanced NSCLC by applying the EuroQol five-dimension scale and scored according to the value set of the Chinese population specific. Disutility associated with AEs was also considered (44–46) (**Supplementary Table 3**).

### Sensitivity analysis

We performed one-way sensitivity analyses and probabilistic sensitivity analyses to test the model’s robustness. In one-way sensitivity analyses, all parameters varied within the 95% confidence interval or ±25% range of the baseline values (when the 95% confidence interval was not available), except for the cost of osimertinib and discount rate (**Supplementary Tables 2**, **3**). Probabilistic sensitivity analyses were conducted using 10,000 Monte Carlo simulations, in which all model inputs varied simultaneously in a certain pattern of distribution. We assumed that costs obey a gamma distribution, probabilities and utilities obey a beta distribution, and HR, BSA, and body weight follow a normal distribution.

### Subgroup analysis

We also incorporated subgroup analyses using the forest plot data from the AURA3 trial and its updated OS analysis (10, 11). Patients were grouped based on race, sex, baseline mutation status, duration of previous EGFR-TKI therapy, CNS metastases at baseline, and smoking history. Due to the insufficient data available, we assumed that all baseline characteristics were consistent between the ITT population and all subgroups, except for the HR values of PFS and OS.

### Scenario analysis

To explore the effect of different WTP threshold levels on the results, we carried out the following scenario analyses: the WTP threshold was changed to $100,000/QALY in the United States (14) and the WTP threshold was varied to $19,003/QALY (three times the per capita gross domestic product of Gansu province in 2021) and $85,176/QALY (three times the per capita gross domestic product of Beijing in 2021) in China (15).

## Results

### Base-case results

In the ITT population, second-line treatment with osimertinib for EGFR T790M advanced NSCLC was associated with an improvement of 0.43 QALYs and incremental $67,588 costs compared with platinum-pemetrexed in the United States, resulting in an ICER of $159,126/QALYs. In China, the osimertinib group yielded an additional 0.34 QALYs with an incremental cost of $1,663, and the ICERs of osimertinib over platinum-pemetrexed was $4,950/QALYs. For patients with CNS metastases, compared with platinum-pemetrexed, the osimertinib strategy provided incremental effectiveness of -0.12 QALYs and -0.14 QALYs with additional costs of $16,465 and $-505 from the perspectives of the United States payers and Chinese healthcare system, respectively, which led to ICERs of $-130,830/QALYs and $3,754/QALYs, respectively. Additional information on the base results is listed in **Table 1**.

**Table 1:** Base case results.

Strategies	China					United States				
	Cost, $	QALYs	LYs	ICER, $/QALY	ICER, $/LY	Cost, $	QALYs	LYs	ICER, $/QALY	ICER, $/LY
ITT population										
Osimertinib	15,748	1.6	2.2	4,950	3,776	224,575	1.75	2.31	159,126	135,017
Platinum-pemetrexed	14,085	1.26	1.76			156,987	1.32	1.81		
Patients with CNS metastases										
Osimertinib	12,860	1.12	1.53	3,754	2,238	169,689	1.2	1.57	-130,830	-69,025
Platinum-pemetrexed	13,365	1.26	1.76			153,224	1.32	1.81		

QALY, quality-adjusted life-years; LYs, life-years; ICER, incremental cost-effectiveness ratio; CNS, central nervous system metastases.

### Sensitivity analysis

**Figure 2:**
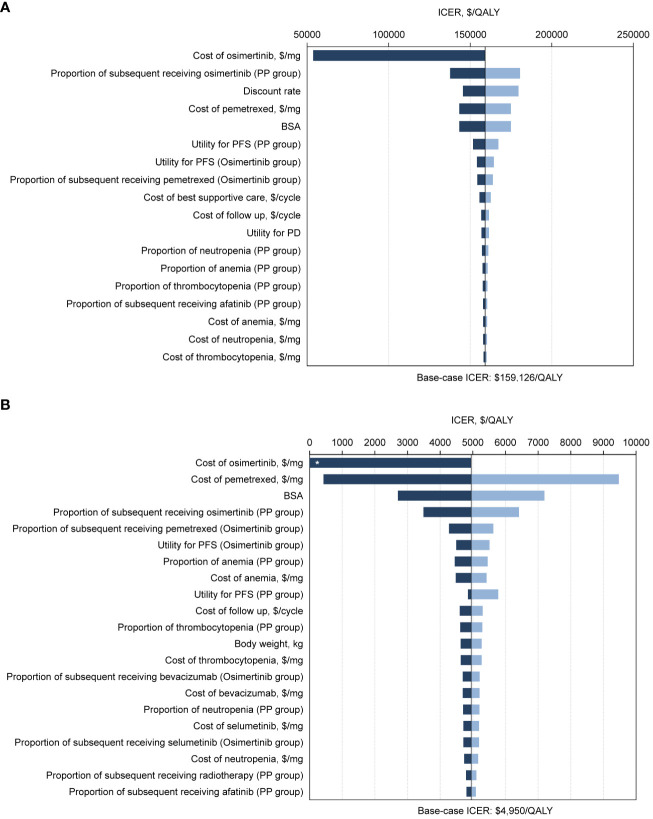
Tornado diagrams of one-way sensitivity analyses with greatest influence variables. The diagram shows the association of variables with the ICER of osimertinib versus platinum-pemetrexed in the second-line treatment of EGFR T790M positive advanced NSCLC in intention-to-treat population, from **(A)** the United States payer and **(B)** Chinese health care system perspectives. The vertical black line represents the base-case result of $159,126 per QALY and $4,950 per QALY in the United States and Chinese context, respectively. *ICER lower than 0. ICER, incremental cost-effectiveness ratio; QALY, quality-adjusted life-years; PP, platinum-pemetrexed; BSA, body surface area; PFS, progression-free survival; PD, progressive disease.

For the ITT population, one-way sensitivity analysis showed that the parameter with the greatest impact on ICERs was the cost of osimertinib in the context of the United States (**Figure 2A**). The cost of osimertinib should be lower than $6.33/mg for osimertinib strategy to be cost-effective. In the Chinese context, the extremely sensitive variables were the cost of osimertinib and the cost of pemetrexed (**Figure 2B**). Within the parameter variation range, no parameter increased the ICERs above the WTP threshold of $37,489 per QALY, and all parameters had only a minor influence.

For patients with CNS metastases, ICERs were most sensitive to the cost of osimertinib in both the United States and Chinese contexts (**Supplementary Figure 4**). In the United States, if the cost of osimertinib drops to $4.90/mg, the osimertinib group would be preferable. In China, osimertinib is the preferred option, regardless of how the cost of osimertinib varies within a given range.

**Figure 3:**
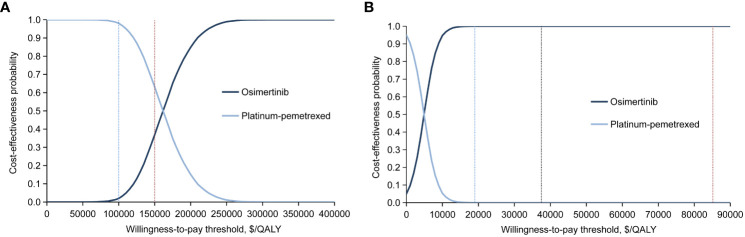
Cost-effectiveness acceptability curves for the osimertinib and platinum-pemetrexed groups in intention-to-treat population generated from the probabilistic sensitivity analysis (10,000 iterations) from **(A)** the United States payer and **(B)** Chinese health care system perspectives. The blue and red vertical dotted line in **(A)** represent the $100,000 and $150,000 per QALY willingness-to-pay thresholds. The blue, black and red vertical dotted line in **(B)** represent the $19,003, $37,489 and $85,176 per QALY willingness-to-pay thresholds. QALY, quality-adjusted life-years.

In probabilistic sensitivity analysis, ICER scatterplot and acceptability curves indicated that for the ITT population, when comparing the osimertinib strategy with platinum-pemetrexed chemotherapy, the probability of second-line osimertinib being cost-effective were 37% and 100%, respectively, at WTP thresholds of $150,000/QALY in the United States and $37,489/QALY in China (**Figure 3**; **Supplementary Figures 5B**, **6B**). For patients with CNS metastases, the osimertinib group had a 5.67% and 26% chance of being cost-effective, with a WTP threshold of $150,000/QALY in the United States and $37,489/QALY in China, respectively (**Supplementary Figures 7B**, **8B**, **9**).

### Subgroup analysis

In the United States, subgroup analysis indicated that the ICERs remained higher at $150,000/QALY in all subgroups except for the “patients without CNS metastases at baseline” subgroup. Probabilistic sensitivity analysis suggested that female sex, smoking history, and non-Asian ethnicity were associated with increased osimertinib cost-effectiveness. In China, osimertinib was most cost-effective in the subgroup without CNS metastases at baseline, followed by female patients, and the duration of previous EGFR-TKI therapy was ≥6 months (**Table 2**).

**Table 2:** Summary of subgroup analyses.

Subgroup	No. of patients	HR of PFS (95% CI)	HR of OS (95% CI)	China				United States			
				Incremental cost, $	Incremental effectiveness,QALY	ICER, $/QALY	Cost-effectiveness probability at WTP ($37,489/QALY)	Incremental cost, $	Incremental effectiveness, QALY	ICER, $/QALY	Cost-effectiveness probability at WTP ($150,000/QALY)
**Race**											
Asian	274	0.32 (0.24-0.44)	0.84 (0.62-1.16)	966	0.21	4,694	90%	53,165	0.26	206,616	25%
Non-Asian	145	0.48 (0.32-0.75)	0.94 (0.63-1.43)	-557	-0.07	-7,452	68%	24,024	0.11	213,345	38%
**Sex**											
Male	150	0.43 (0.28-0.65)	1.11 (0.72-1.76)	-1,080	-0.08	13,132	39%	14,719	-0.06	-240,880	17%
Female	269	0.34 (0.25-0.47)	0.77 (0.57-1.05)	1,268	0.33	4,182	98%	58,780	0.37	160,312	43%
**Baseline mutation status**											
Exon 19 deletion	279	0.34 (0.24-0.46)	0.88 (0.64-1.22)	566	0.15	3,698	84%	45,608	0.2	229,296	23%
L858R	128	0.46 (0.30-0.71)	0.96 (0.64-1.46)	-562	0.05	-10,316	65%	24,002	0.09	266,436	32%
**Duration of previous EGFR-TKI therapy**											
<6 mo^a^	24	–	–	–	–	–	–	–	–	–	–
≥6 mo	395	0.39 (0.30-0.51)	0.87 (0.68-1.13)	286	0.16	1,769	92%	40,117	0.21	192,366	32%
**CNS metastases at baseline**											
Yes	144	0.32 (0.21-0.49)	1.19 (0.79-1.83)	-505	-0.14	3,754	26%	16,465	-0.12	-130,830	5.67%
No	275	0.40 (0.29-0.55)	0.75 (0.55-1.03)	978	0.33	2,969	96%	53,234	0.4	134,186	61%
**Smoking history**											
Yes	136	0.40 (0.27-0.62)	0.87 (0.55-1.40)	225	0.16	1,399	77%	38,939	0.21	187,370	39%
No	283	0.36 (0.26-0.49)	0.87 (0.65-1.18)	482	0.16	2,942	87%	43,917	0.21	208,251	28%

a As the number of patients with duration of previous EGFR-TKI therapy <6 months was small and not well represented in the model, we excluded them from our subgroup analysis.

HR, hazard ratio; CNS, central nervous system metastases; HR, hazard ratio; PFS, progression-free survival; OS, overall survival; ICER, incremental cost-effectiveness ratio; QALY, quality-adjusted life-years; EGFR, epidermal growth factor receptor; TKI, tyrosine kinase inhibitor.

### Scenario analysis

In the United States, at a WTP threshold of $100,000/QALY, the probability of osimertinib being cost-effectiveness was 2% (**Figure 3A**; **Supplementary Figure 5A**) and 1.02% (**Supplementary Figures 7A**, **9A**) for the ITT population and CNS metastases population, respectively, significantly lower than that of 37% and 5.67% at the base case scenario (WTP threshold of $150,000/QALY).

In China, there was no effect on the probability of osimertinib being cost-effective when the WTP threshold varied in the range of $19,003/QALY to $85,176/QALY. For the ITT population, when the WTP threshold was greater than $18,243/QALY, the probability of osimertinib being cost-effective was always 100% (**Figure 3B** and **Supplementary Figures 6A, C**). For patients with CNS metastases, when the WTP threshold was greater than $14,129/QALY, the probability of osimertinib being cost-effectiveness was always about 26% (**Supplementary Figures 8A, C**, **9B**).

## Discussion

Given the recent release of mature OS data from the AURA3 trial, the cost-effectiveness of osimertinib in second-line treatment should be updated accordingly. This analysis estimated the costs and effectiveness of osimertinib second-line treatment in the ITT population as well as in patients with CNS metastases, in both developed and developing countries. In the United States, for ITT patients, osimertinib was not a cost-effective strategy at a WTP threshold of $150,000 per QALY because it provided an additional cost of $67,588. The variable that extremely sensitive to ICERs was the cost of osimertinib. We found that osimertinib dominates platinum-pemetrexed economically when the cost of osimertinib decreases to $6.33/mg. For patients with CNS metastases, osimertinib is costlier and less effective than platinum-pemetrexed at the current WTP ($150,000/QALY). Osimertinib would be a cost-effective option when its price decreases to $4.90/mg. In China, for ITT patients, osimertinib is the dominant strategy compared with platinum-pemetrexed at a WTP threshold of $37,489 and remains the preferred strategy, regardless of how all model parameters change within a given range. For patients with CNS metastases, osimertinib is less effective; however, its costs are lower, and ICERs ($3,754 per QALY) is lower than the WTP threshold, making it a preferred option. Subgroup characteristics associated with cost-effectiveness suggest that the absence of CNS metastases at baseline is the most important factor for osimertinib cost-effectiveness. Scenario analysis showed that in the United States, the probability of osimertinib being cost-effectiveness was significantly lower when the WTP threshold was reduced from $150,000/QALY to $100,000/QALY for both the ITT population and the CNS metastases population. This suggests that osimertinib is more economical in areas with higher income per capita in the United States. In China, the probability of osimertinib being cost-effectiveness remained constant at 100% and 26% for the ITT population and CNS metastases populations, respectively, when the WTP threshold was $19,003/QALY, $37,489/QALY, and $85,176/QALY (Representing the regions with the lowest GDP per capita, the national average GDP per capita and the highest GDP per capita, respectively). This indicates that the cost-effectiveness of osimertinib in China is consistent across areas of different economic backgrounds.

Two prior studies by Wu et al. (47) and Guan H et al. (44) assessed the economics of second-line osimertinib and chemotherapy in patients with EGFR T790M NSCLC, from the perspectives of the United States and China. In the analysis of Wu et al. (47), osimertinib has no cost-effectiveness in either the ITT population or in CNS metastases, in both the contexts of the United States and China. Guan et al. (44) suggested that osimertinib is a cost-effective option for ITT patients in the context of China. There are differences between our analysis and those of Wu et al. and Guan et al. that should be noted. First, immature survival data were used in the two prior studies, due to the OS data of AURA3 being unavailable. Mature and reliable data is the basis for a robust model. In the model of Wu et al., the post-progression survival data were based on a systematic review (48) of third-line chemotherapy in advanced NSCLC. The assumption that the post-progression survival was the same in the osimertinib and chemotherapy arms was inconsistent with the results in the AURA3 OS analysis (11) and may result in overestimation of the health benefits of osimertinib. The OS data associated with osimertinib in the Guan et al. (44) model were less mature and derived from the pooled result of two single-arm phases II studies (49), in which not all patients received second-line treatment. The OS data on platinum-pemetrexed was obtained from the IMPRESS trial (50), in which the median OS of 14.1 months was significantly lower than the final OS results reported in the OS analysis of the AURA3 trial (11) (median OS 22.5 months). This may have led to an underestimation of the OS benefits of the two strategies. Secondly, the health utilities for the United States population in Wu et al.’s study were derived from study data of the health utilities of populations in the United Kingdom. Notably, the health preferences of populations in different countries and regions vary significantly (42, 45). Thus, the utilities based on the United Kingdom cannot accurately reflect health preferences outside the United Kingdom context. Lastly, the current price of osimertinib ($0.36/mg) in China has been reduced by 89% through the National Reimbursement Drug List negotiation in March 2021. This has a potential impact on the economics of osimertinib because the price of the drug was the most sensitive factor to model robustness according to Wu et al. and Guan et al. (44, 47).

To our knowledge, this is the first economic evaluation of second-line osimertinib in both ITT and CNS metastases patients, based on the latest evidence from the AURA3 trial and the most recent reimbursement prices of osimertinib in China (11). This study had several strengths. First, the latest evidence in the AURA3 trial, a well-conducted phase III clinical trial, was synthesized in this study (11). Second, we considered AE-related treatment discontinuation rates of osimertinib and platinum-pemetrexed. Third, we performed subgroup analyses to assess the economy of patients with different baseline characteristics, based on the forest plot of the AURA3 trial (10, 11).

Economic evaluations are widely adopted methods for assessing value and affordability, and have become an essential part of the pricing and reimbursement process of new interventions in many countries such as the United Kingdom, Australia, Canada, and so on (51). Osimertinib is much more expensive in the United States ($6.62/mg) than in China ($0.36/mg) because of the differences in prescription and reimbursement policies. In the United States, Medicare, the biggest insurer, covers almost all launched cancer drugs, which greatly limits negotiations with the producer (52). So that the pricing of cancer drugs often fails to reflect the innovation and efficacy of the drugs (53). However, in recent years, with the increasing cost of health care, especially in cancer treatment, many institutions and medical professional societies in the United States, such as the American Society of Clinical Oncology, the National Comprehensive Cancer Network, and the European Society for Medical Oncology are assigning more importance to develop value-based frameworks of novel interventions to consider not only safety and efficacy but also the economy (54, 55). Reducing the price of cancer drugs through tradeoff negotiations on drug prices and coverage may be an effective way to improve cost-effectiveness. In China, price negotiation mechanisms involving pharmaceutical companies and other stakeholders for patented drugs and exclusive drugs were implemented in 2015 (56). The Interim Measures for the Administration of Drugs under Basic Medical Insurance issued in July 2020 requires that the economic evaluation must be submitted for drugs movement into or out of the National Reimbursement Drug List and for some drugs whose limited scope of payment is expanded (57). The pharmacoeconomic evaluation results have become an important factor in the negotiation of drug access in the national reimbursement drug list. During the negotiations between the National Healthcare Security Administration and producers in 2018 and 2021, osimertinib has gone through two rounds of price cuts that have reduced its price by 89%, from the original $3.40/mg to $0.36/mg. This greatly reduces the financial burden on patients with tumors in China.

This study has some limitations. First, the data of our model is solely from the AURA3 trial, which may be biased. However, the AURA3 trial is a multicenter, well-designed phase III clinical trial that investigates second-line osimertinib and platinum-pemetrexed in EGFR T790M advanced NSCLC. Second, in the absence of OS Kaplan-Meier survival curves of the CNS metastases population, we assumed that the OS rate of the platinum-pemetrexed group was the same in patients with CNS metastases and the ITT population. Notably, the median OS in patients with CNS metastases was consistent with that in the ITT population according to the AURA3 study (11). Third, due to the lack of a Kaplan-Meier curve for each subgroup in the AURA3 trial, we assumed that all baseline characteristics were consistent between the ITT population and all subgroups, except for the HR values of PFS and OS. Thus, the results of the subgroup analysis should be interpreted with caution. Additionally, the subgroup sample size was small, which affected the robustness of the model. Fourth, the options for subsequent treatment after disease progression in both treatment groups were sourced from the AURA3 trial, which might be biased against the real-world clinical practice in the United States and China. We performed one-way and probabilistic sensitivity analyses to address this uncertainty.

Our findings indicated that osimertinib is not cost-effective compared to platinum-pemetrexed in second-line treatment in EGFR T790M advanced NSCLC, from the United States payer standpoint. Lowering the price of osimertinib is the most practical measure to make second-line osimertinib treatment cost-effective. From the Chinese healthcare system perspective, osimertinib is the dominant treatment strategy compared with platinum-pemetrexed in the ITT population and provides additional cost savings in patients with CNS metastases, regardless of the level of per capita income.

## Data availability statement

The original contributions presented in the study are included in the article/**Supplementary Material**. Further inquiries can be directed to the corresponding author.

## Author contributions

YS: Conceptualization, Methodology, Validation, Formal analysis, Writing - original draft. RP: Software, Formal analysis, Investigation. SL: Resources, Writing - review & editing, Supervision, Project administration. All authors read and approved the final manuscript.

## Funding

This work was supported by the Hunan Provincial Natural Science Foundation [grant 2022JJ70071].

## Conflict of interest

The authors declare that the research was conducted in the absence of any commercial or financial relationships that could be construed as a potential conflict of interest.

## Publisher’s note

All claims expressed in this article are solely those of the authors and do not necessarily represent those of their affiliated organizations, or those of the publisher, the editors and the reviewers. Any product that may be evaluated in this article, or claim that may be made by its manufacturer, is not guaranteed or endorsed by the publisher.
