# Supplementary material for: Osimertinib versus platinum-pemetrexed in patients with previously treated EGFR T790M advanced non-small cell lung cancer: An updated AURA3 trial-based cost-effectiveness analysis
Source: Front Oncol. 2022 Oct 17;12:833773. doi: 10.3389/fonc.2022.833773 (PMC9619214; doi:10.3389/fonc.2022.833773)
Supplement: Supplementary file 1 [file DataSheet_1.pdf]

Supplementary Material

Supplementary Figure 1. PFS Kaplan-Meier curve of intention-to-treat population fitting and extrapolation

Supplementary Figure 2. OS Kaplan-Meier curve of intention-to-treat population fitting and extrapolation

Supplementary Figure 3. PFS Kaplan-Meier curve of patients with CNS metastases fitting and extrapolation. CNS, central nervous system metastases

Supplementary Figure 4. Tornado diagrams of one-way sensitivity analyses with the greatest influence variables

Supplementary Figure 5. Incremental cost-effectiveness scatter plot for the osimertinib and platinum-pemetrexed groups in intention-to-treat population generated from the probabilistic sensitivity analysis (10,000 iterations) from the United States payer perspectives. (A) WTP was $100,000/QALY (B) WTP was $150,000/QALY. QALYs, quality-adjusted life-years; WTP, willingness-to-pay

Supplementary Figure 6. Incremental cost-effectiveness scatter plot for the osimertinib and platinum-pemetrexed groups in intention-to-treat population generated from the probabilistic sensitivity analysis (10,000 iterations) from Chinese health care system perspectives. (A) WTP was $19,003/QALY, (B) WTP was $37,489/QALY, (C) WTP was $85,176/QALY. QALYs, quality-adjusted life-years; WTP, willingness-to-pay

Supplementary Figure 7. Incremental cost-effectiveness scatter plot for the osimertinib and platinum-pemetrexed groups in patients with CNS metastases generated from the probabilistic sensitivity analysis (10,000 iterations) from the United States payer perspectives. (A) WTP was $100,000/QALY (B) WTP was $150,000/QALY. CNS, central nervous system metastases; QALYs, quality-adjusted life-years; WTP, willingness-to-pay

Supplementary Figure 8. Incremental cost-effectiveness scatter plot for the osimertinib and platinum-pemetrexed groups in patients with CNS metastases generated from the probabilistic sensitivity analysis (10,000 iterations) from Chinese health care system perspectives. (A) WTP was $19,003/QALY, (B) WTP was $37,489/QALY, (C) WTP was $85,176/QALY. CNS, central nervous system metastases; QALYs, quality-adjusted life-years; WTP, willingness-to-pay

Supplementary Figure 9. Cost-effectiveness acceptability curves for the osimertinib and platinum-pemetrexed groups in patients with CNS metastases generated from the probabilistic sensitivity analysis (10,000 iterations) from **(A)** the United States payer and **(B)** Chinese health care system perspectives. The blue and red vertical dotted line in Supplementary Figure 9A represent the $100,000 and $150,000 per QALY willingness-to-pay thresholds. The blue, black and red vertical dotted line in Supplementary Figure 9B represent the $19,003, $37,489 and $85,176 per QALY willingness-to-pay thresholds. CNS, central nervous system metastases; QALYs, quality-adjusted life-years

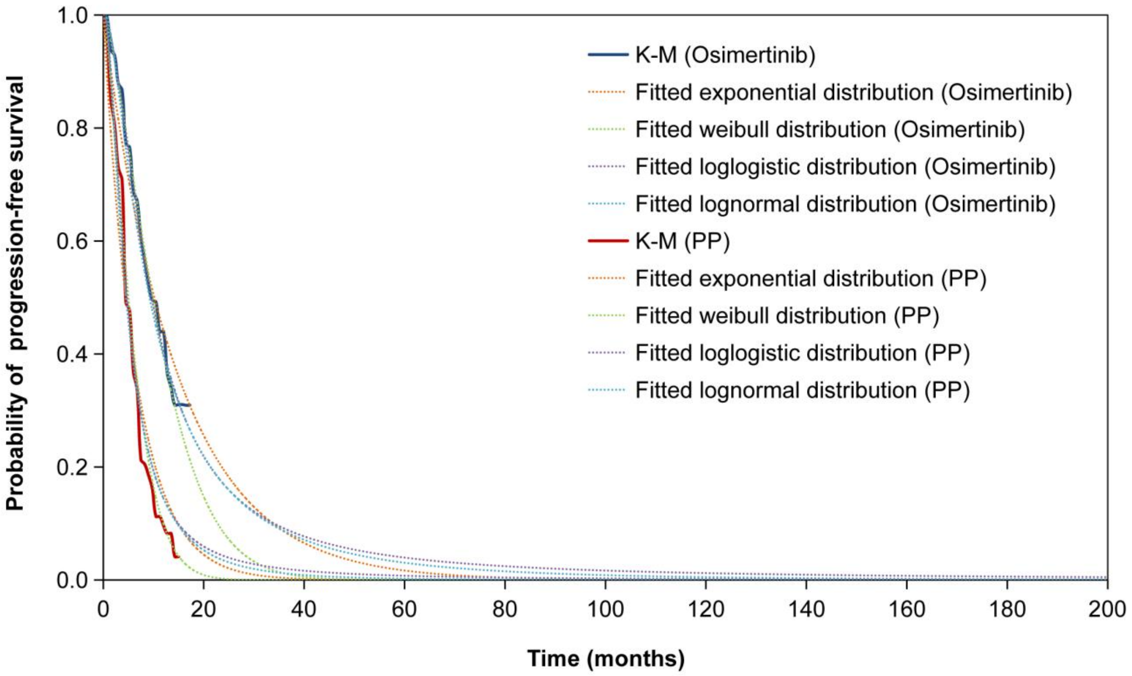

Supplementary Figure 1. PFS Kaplan-Meier curve in intention-to-treat fitting and extrapolation. PFS, progression-free survival; PP, Platinum-pemetrexed

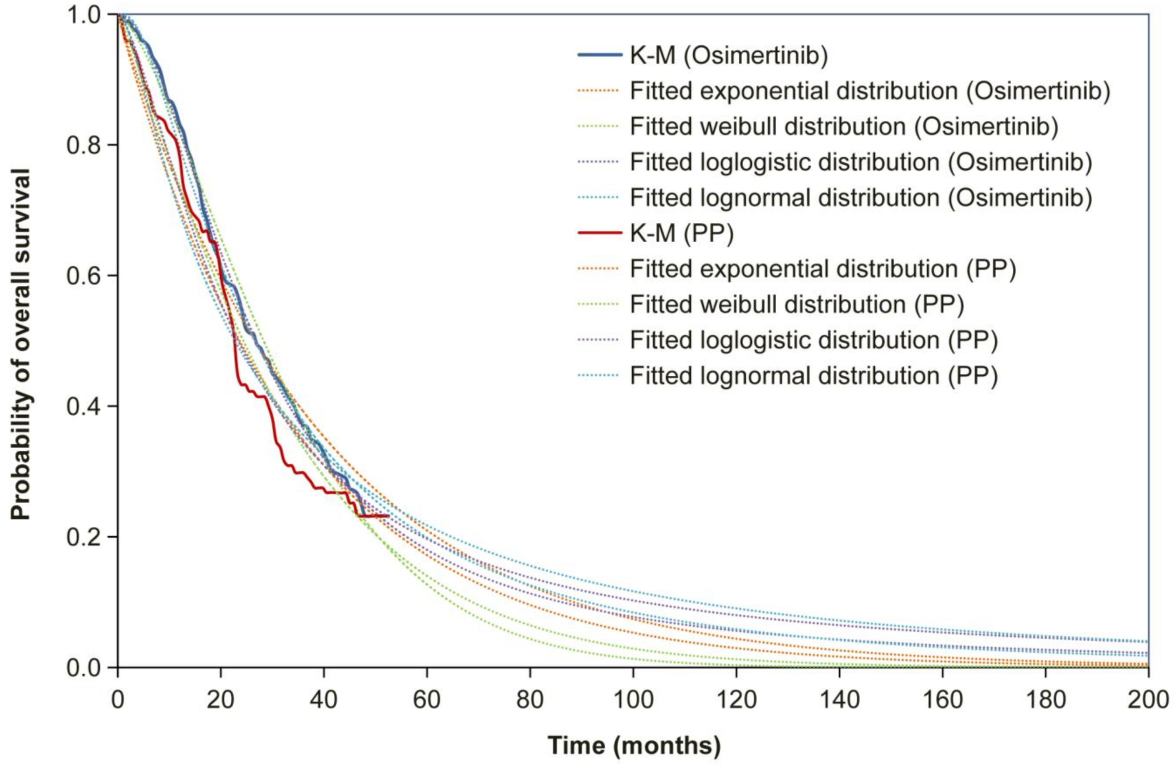

Supplementary Figure 2. OS Kaplan-Meier curve in intention-to-treat population fitting and extrapolation. OS, overall survival; PP, Platinum-pemetrexed

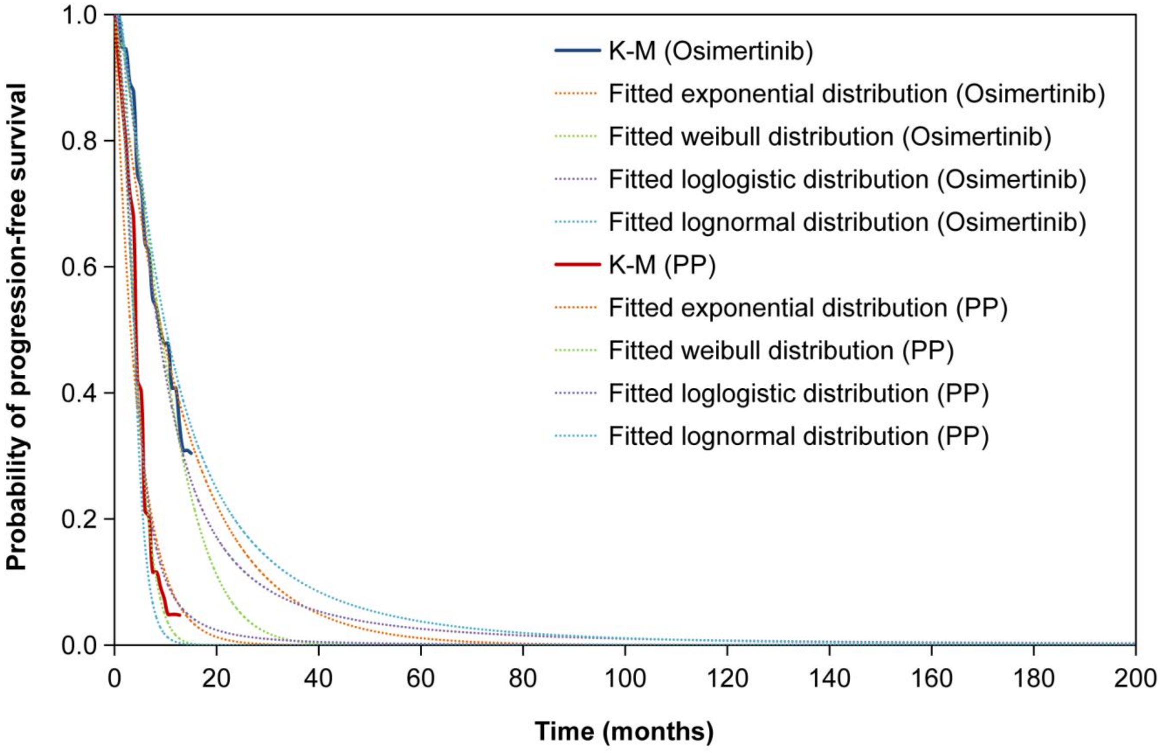

Supplementary Figure 3. PFS Kaplan-Meier curve in patients with CNS metastases fitting and extrapolation. PFS, progression-free survival; PP, Platinum-pemetrexed

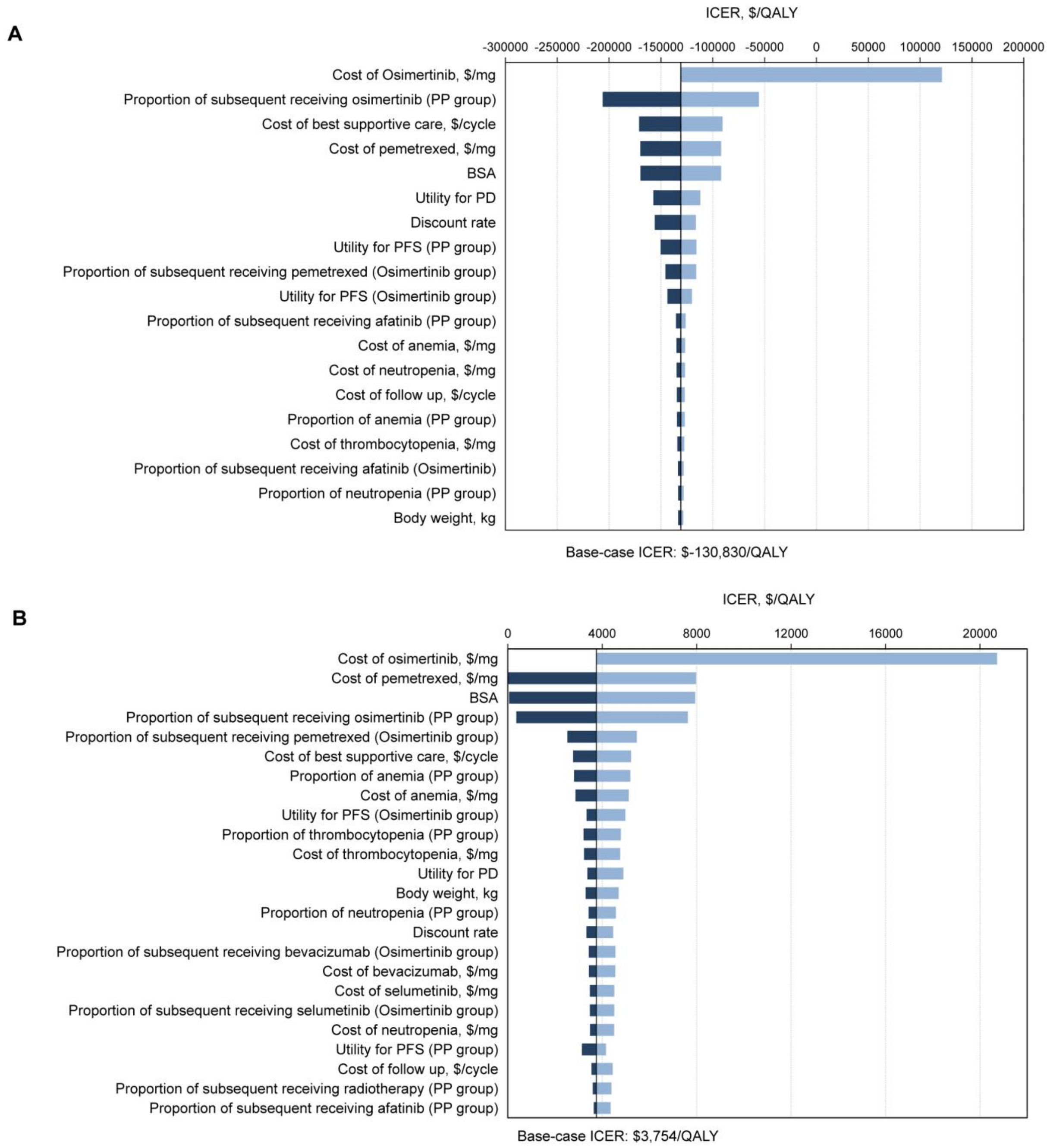

Supplementary Figure 4. Tornado diagrams of one-way sensitivity analyses with the greatest influence variables. The diagram shows the association of variables with the ICER of osimertinib versus platinum-pemetrexed in the second-line treatment of EGFR T790M positive advanced NSCLC in patients with CNS metastases, from **(A)** the United States payer and **(B)** Chinese health care system perspectives. The vertical black line represents the base-case result of $-130,830 per QALY and $3,754 per QALY in the United States and Chinese context, respectively. ICER, incremental cost-effectiveness ratio; QALY, quality-adjusted life-years; PP, platinum-pemetrexed; BSA, body surface area; PFS, progression-free survival; PD, progressive disease

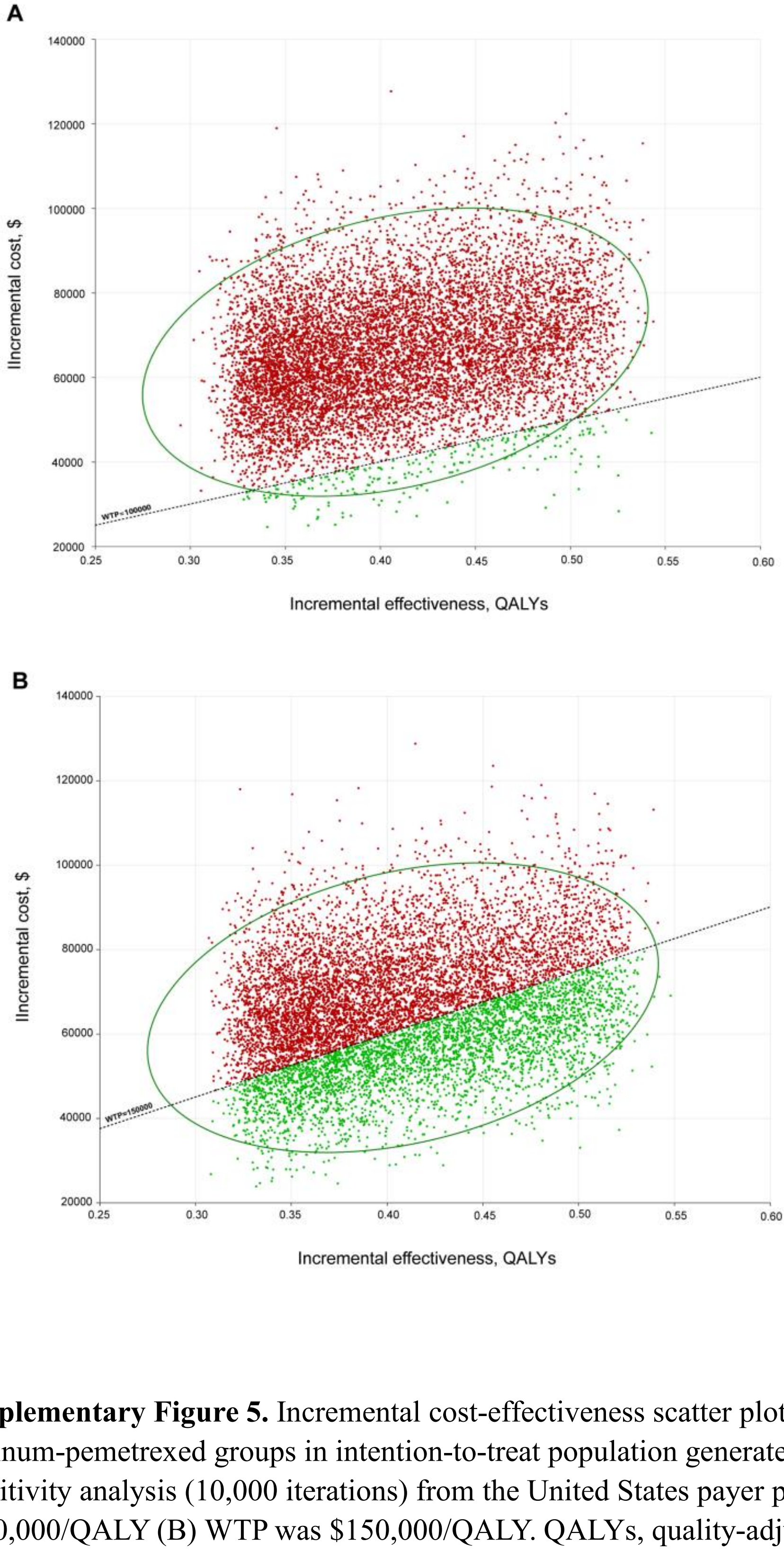

Supplementary Figure 5. Incremental cost-effectiveness scatter plot for the osimertinib and platinum-pemetrexed groups in intention-to-treat population generated from the probabilistic sensitivity analysis (10,000 iterations) from the United States payer perspectives. (A) WTP was $100,000/QALY (B) WTP was $150,000/QALY. QALYs, quality-adjusted life-years; WTP, willingness-to-pay

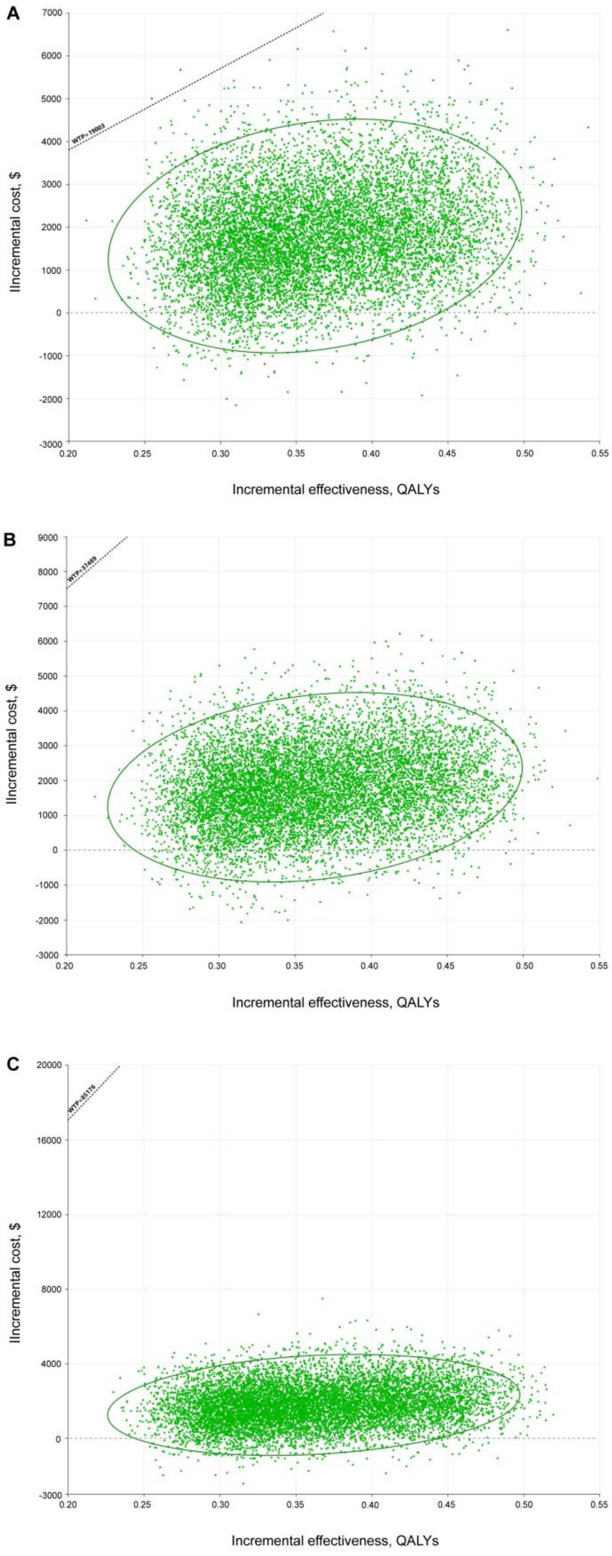

Supplementary Figure 6. Incremental cost-effectiveness scatter plot for the osimertinib and platinum-pemetrexed groups in intention-to-treat population generated from the probabilistic sensitivity analysis (10,000 iterations) from Chinese health care system perspectives. (A) WTP was $19,003/QALY, (B) WTP was $37,489/QALY, (C) WTP was $85,176/QALY. QALYs, quality-adjusted life-years; WTP, willingness-to-pay

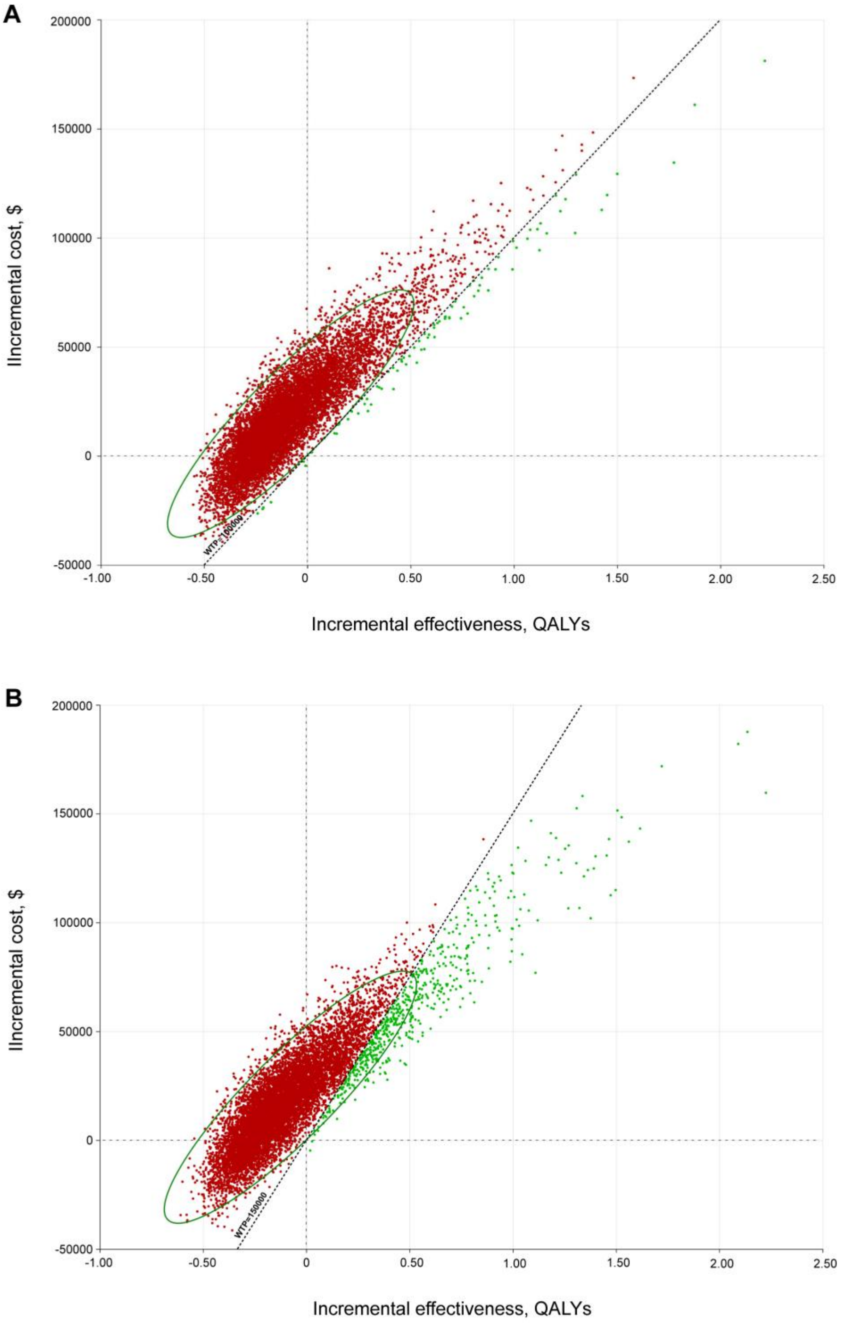

Supplementary Figure 7. Incremental cost-effectiveness scatter plot for the osimertinib and platinum-pemetrexed groups in patients with CNS metastases generated from the probabilistic sensitivity analysis (10,000 iterations) from the United States payer perspectives. (A) WTP was $100,000/QALY (B) WTP was $150,000/QALY. CNS, central nervous system metastases; QALYs, quality-adjusted life-years; WTP, willingness-to-pay

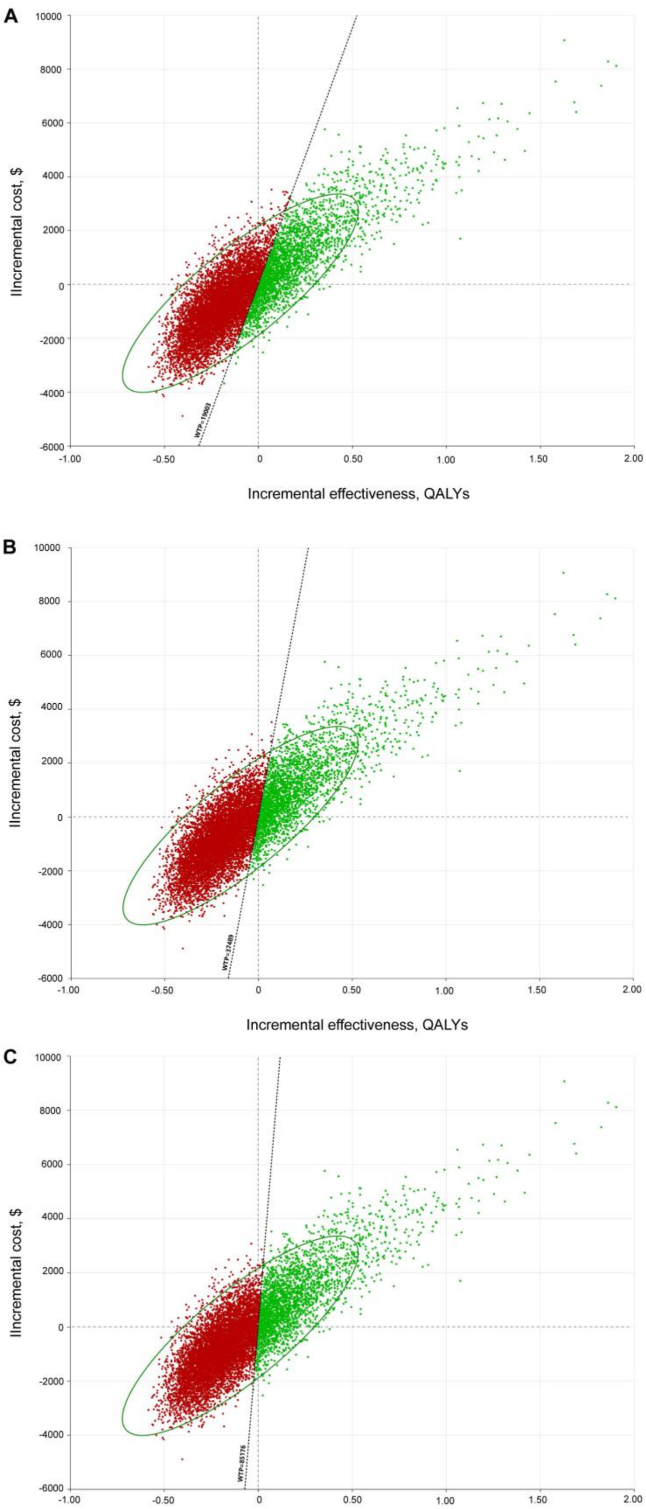

Supplementary Figure 8. Incremental cost-effectiveness scatter plot for the osimertinib and platinum-pemetrexed groups in patients with CNS metastases generated from the probabilistic sensitivity analysis (10,000 iterations) from Chinese health care system perspectives. (A) WTP was $19,003/QALY, (B) WTP was $37,489/QALY, (C) WTP was $85,176/QALY. CNS, central nervous system metastases; QALYs, quality-adjusted life-years; WTP, willingness-to-pay

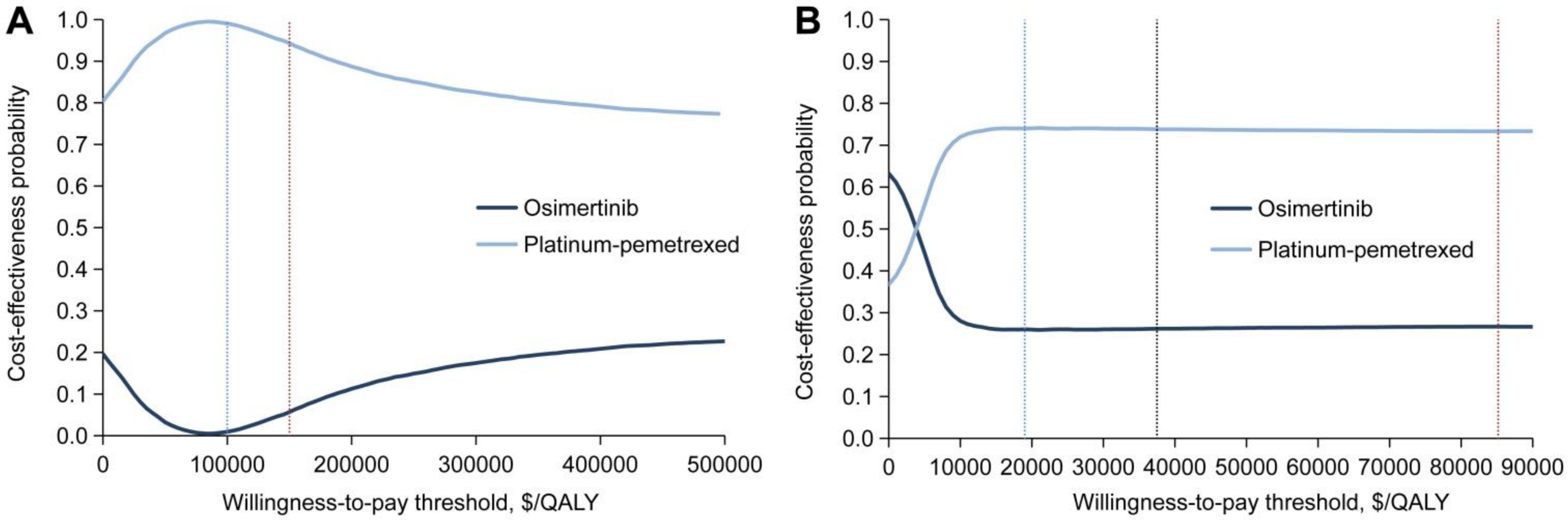

Supplementary Figure 9. Cost-effectiveness acceptability curves for the osimertinib and platinum-pemetrexed groups in patients with CNS metastases generated from the probabilistic sensitivity analysis (10,000 iterations) from **(A)** the United States payer and **(B)** Chinese health care system perspectives. The blue and red vertical dotted line in Supplementary Figure 9A represent the $100,000 and $150,000 per QALY willingness-to-pay thresholds. The blue, black and red vertical dotted line in Supplementary Figure 9B represent the $19,003, $37,489 and $85,176 per QALY willingness-to-pay thresholds. CNS, central nervous system metastases; QALYs, quality-adjusted life-years
