# Supplementary material for: Osimertinib versus platinum-pemetrexed in patients with previously treated EGFR T790M advanced non-small cell lung cancer: An updated AURA3 trial-based cost-effectiveness analysis
Source: Front Oncol. 2022 Oct 17;12:833773. doi: 10.3389/fonc.2022.833773 (PMC9619214; doi:10.3389/fonc.2022.833773)
Supplement: Supplementary file 2 [file DataSheet_2.docx]

**Supplementary Material**

**Supplementary Table 1.** Patient Baseline Characteristics

**Supplementary Table 2.** Key Clinical Data

**Supplementary Table 3.** Base-Case Cost Estimates and Utilities

**Supplementary Table 4.** R statistics code: output for the common parametric survival distributions.

**Supplementary Table 5.** Summary of statistical goodness-of-fit of Kaplan-Meier curve in the AURA3 trial

**Supplementary Table 6.** CHEERS checklist

**Supplementary References**

**Supplementary Table 1.** Patient Baseline Characteristics.

| **Characteristics** | **Osimertinib**  **(n=279)** | **Platinum-pemetrexed (n=140)** |
| --- | --- | --- |
| Median Age (range) — years | 62 (25-85) | 63 (20-90) |
| Female sex — no. (%) | 172 (62) | 97 (69) |
| Race — no. (%) |  |  |
| Asian | 182 (65) | 92 (66) |
| Non-Asian | 97 (35) | 48 (34) |
| No history of smoking — no. (%) | 189 (68) | 94 (67) |
| Disease classification — no. (%) |  |  |
| Adenocarcinoma histology not otherwise specified | 232 (83) | 122 (87) |
| Metastatic disease | 266 (95) | 138 (99) |
| CNS metastases | 93 (33) | 51 (36) |
| Extrathoracic visceral metastases | 145 (52) | 80 (57) |
| Type of EGFR mutation — no. (%) |  |  |
| T790M | 275 (99) | 138 (99) |
| Exon 19 deletion | 191 (68) | 87 (62) |
| Exon 21 L858R | 83 (30) | 45 (32) |
| G719X | 4 (1) | 2 (1) |
| S768I | 1 (<1) | 1 (1) |
| Exon 20 insertion | 1 (<1) | 2 (1) |
| No. of previous anticancer regimens for advanced disease — no. (%) |  |  |
| 1 | 269 (96) | 134 (96) |
| 2 | 9 (3) | 6 (4) |
| 3 | 1 (<1) | 0 |
| Previous EGFR-TKI therapy — no. (%) |  |  |
| Gefitinib | 166 (59) | 87 (62) |
| Erlotinib | 96 (34) | 49 (35) |
| Afatinib | 20 (7) | 4 (3) |

The baseline characteristics of the hypothetical patients in the model were derived from the relevant data of AURA3 trial (1). CNS, central nervous system; EGFR, epidermal growth factor receptor; TKI, tyrosine kinase inhibitor

**Supplementary Table 2.** Key Clinical Data.

| **Parameter** | **United States** | | | | | **China** | | | | |
| --- | --- | --- | --- | --- | --- | --- | --- | --- | --- | --- |
|  | **Values** | **Range** | |  |  | **Values** | **Range** | |  |  |
|  |  | **Lower limit** | **Upper limit** | **Distribution (Parameters)** | **Reference** |  | **Lower limit** | **Upper limit** | **Distribution (Parameters)** | **Reference** |
| Weibull survival model of  platinum-pemetrexed (ITT population) |  |  |  |  |  |  |  |  |  |  |
| PFS | λ=0.0738  γ=1.3873 | - | - | - | Model fitting | λ=0.0738  γ=1.3873 | - | - | - | Model fitting |
| OS | λ=0.0173  γ=1.1565 | - | - | - | Model fitting | λ=0.0173  γ=1.1565 | - | - | - | Model fitting |
| Log-logistic survival model of osimertinib (ITT population) |  |  |  |  |  |  |  |  |  |  |
| PFS | λ=0.0194  γ=1.7414 | - | - | - | Model fitting | λ=0.0194  γ=1.7414 | - | - | - | Model fitting |
| OS | λ=0.0020  γ=1.8850 | - | - | - | Model fitting | λ=0.0020  γ=1.8850 | - | - | - | Model fitting |
| Weibull survival model of  platinum-pemetrexed (Patients with CNS metastases) |  |  |  |  |  |  |  |  |  |  |
| PFS | λ=0.0766  γ=1.6080 | - | - | - | Model fitting | λ=0.0766  γ=1.6080 | - | - | - | Model fitting |
| Log-logistic survival model of  osimertinib (Patients with CNS metastases) |  |  |  |  |  |  |  |  |  |  |
| PFS | λ=0.0182  γ=1.8651 | - | - | - | Model fitting | λ=0.0182  γ=1.8651 | - | - | - | Model fitting |
| HR of osimertinib versus platinum-pemetrexed for OS (Patients with CNS metastases) | 1.19 | 0.79 | 1.83 | Normal (1.19, 0.27) | (2) | 1.19 | 0.79 | 1.83 | Normal (1.19, 0.27) | (2) |
| AEs (≥ grade 3) incidences |  |  |  |  |  |  |  |  |  |  |
| Osimertinib group |  |  |  |  |  |  |  |  |  |  |
| Neutropenia | 0.004 | 0.003 | 0.005 | Beta (61.22, 15242.72) | (2) | 0.004 | 0.003 | 0.005 | Beta (61.22, 15242.72) | (2) |
| Anemia | 0.004 | 0.003 | 0.005 | Beta (61.22, 15242.72) | (2) | 0.004 | 0.003 | 0.005 | Beta (61.22, 15242.72) | (2) |
| Thrombocytopenia | 0 | 0.000 | 0.000 | - | (2) | 0 | 0.000 | 0.000 | - | (2) |
| Platinum-pemetrexed group |  |  |  |  |  |  |  |  |  |  |
| Neutropenia | 0.110 | 0.083 | 0.138 | Beta (54.59, 441.72) | (2) | 0.110 | 0.083 | 0.138 | Beta (54.59, 441.72) | (2) |
| Anemia | 0.096 | 0.072 | 0.120 | Beta (55.47, 522.33) | (2) | 0.096 | 0.072 | 0.120 | Beta (55.47, 522.33) | (2) |
| Thrombocytopenia | 0.066 | 0.050 | 0.083 | Beta (57.34, 811.49) | (2) | 0.066 | 0.050 | 0.083 | Beta (57.34, 811.49) | (2) |
| The proportion of subsequent therapy ^a^ |  |  |  |  |  |  |  |  |  |  |
| Osimertinib group |  |  |  |  |  |  |  |  |  |  |
| Osimertinib | 0 | 0.000 | 0.000 | - |  | 0 | 0.000 | 0.000 | - |  |
| EGFR protein kinase inhibitors (other than osimertinib) | 0.065 | 0.049 | 0.081 | Beta (57.41, 825.75) | (2) | 0.065 | 0.049 | 0.081 | Beta (57.41, 825.75) | (2) |
| Pemetrexed | 0.391 | 0.293 | 0.489 | Beta (37.04, 57.69) | (2) | 0.391 | 0.293 | 0.489 | Beta (37.04, 57.69) | (2) |
| Cytotoxic chemotherapy platinum compounds | 0.387 | 0.290 | 0.484 | Beta (37.29, 59.07) | (2) | 0.387 | 0.290 | 0.484 | Beta (37.29, 59.07) | (2) |
| Cytotoxic chemotherapy taxanes | 0.050 | 0.038 | 0.063 | Beta (58.34, 1108.50) | (2) | 0.050 | 0.038 | 0.063 | Beta (58.34, 1108.50) | (2) |
| Antibody against VEGF | 0.050 | 0.038 | 0.063 | Beta (58.34, 1108.50) | (2) | 0.050 | 0.038 | 0.063 | Beta (58.34, 1108.50) | (2) |
| Cytotoxic chemotherapy  podophyllotoxin derivative | 0.018 | 0.014 | 0.023 | Beta (60.34, 3291.95) | (2) | 0.018 | 0.014 | 0.023 | Beta (60.34, 3291.95) | (2) |
| MET inhibitor | 0.018 | 0.014 | 0.023 | Beta (60.34, 3291.95) | (2) | 0.018 | 0.014 | 0.023 | Beta (60.34, 3291.95) | (2) |
| Cytotoxic chemotherapy vinca alkaloids and analogs | 0.011 | 0.008 | 0.014 | Beta (60.78, 5464.54) | (2) | 0.011 | 0.008 | 0.014 | Beta (60.78, 5464.54) | (2) |
| Radiotherapy | 0.004 | 0.003 | 0.005 | Beta (61.22,15242.72) | (2) | 0.004 | 0.003 | 0.005 | Beta (61.22,15242.72) | (2) |
| MEK inhibitor | 0.007 | 0.005 | 0.009 | Beta (61.03, 8657.31) | (2) | 0.007 | 0.005 | 0.009 | Beta (61.03, 8657.31) | (2) |
| Unknown | 0.007 | 0.005 | 0.009 | Beta (61.03, 8657.31) | (2) | 0.007 | 0.005 | 0.009 | Beta (61.03, 8657.31) | (2) |
| Antibody against PD-1 | 0.007 | 0.005 | 0.009 | Beta (61.03, 8657.31) | (2) | 0.007 | 0.005 | 0.009 | Beta (61.03, 8657.31) | (2) |
| Antibody against EGFR | 0.004 | 0.003 | 0.005 | Beta (61.22,15242.72) | (2) | 0.004 | 0.003 | 0.005 | Beta (61.22,15242.72) | (2) |
| Antibody against HER2 | 0.004 | 0.003 | 0.005 | Beta (61.22,15242.72) | (2) | 0.004 | 0.003 | 0.005 | Beta (61.22,15242.72) | (2) |
| Cytotoxic chemotherapy | 0.004 | 0.003 | 0.005 | Beta (61.22,15242.72) | (2) | 0.004 | 0.003 | 0.005 | Beta (61.22,15242.72) | (2) |
| Platinum-pemetrexed group |  |  |  |  |  |  |  |  |  |  |
| Osimertinib | 0.700 | 0.525 | 0.875 | Beta (17.74, 7.60) | (2) | 0.700 | 0.525 | 0.875 | Beta (17.74, 7.60) | (2) |
| EGFR protein kinase inhibitors (other than osimertinib) | 0.064 | 0.048 | 0.080 | Beta (57.47, 840.47) | (2) | 0.064 | 0.048 | 0.080 | Beta (57.47, 840.47) | (2) |
| Pemetrexed | 0.014 | 0.011 | 0.018 | Beta (60.59, 4267.34) | (2) | 0.014 | 0.011 | 0.018 | Beta (60.59, 4267.34) | (2) |
| Cytotoxic chemotherapy platinum compounds | 0.007 | 0.005 | 0.009 | Beta (61.03, 8657.31) | (2) | 0.007 | 0.005 | 0.009 | Beta (61.03, 8657.31) | (2) |
| Cytotoxic chemotherapy taxanes | 0.007 | 0.005 | 0.009 | Beta (61.03, 8657.31) | (2) | 0.007 | 0.005 | 0.009 | Beta (61.03, 8657.31) | (2) |
| Radiotherapy | 0.007 | 0.005 | 0.009 | Beta (61.03, 8657.31) | (2) | 0.007 | 0.005 | 0.009 | Beta (61.03, 8657.31) | (2) |
| Rate of treatment discontinuation due to AEs |  |  |  |  |  |  |  |  |  |  |
| Osimertinib group | 0.07 | 0.053 | 0.088 | Beta (57.16, 759.45) | (1) | 0.07 | 0.053 | 0.088 | Beta (57.16, 759.45) | (1) |
| Platinum-pemetrexed group | 0.10 | 0.075 | 0.125 | Beta (55.32, 497.87) | (1) | 0.10 | 0.075 | 0.125 | Beta (55.32, 497.87) | (1) |
| BSA (m^2^) | 1.79 | 1.34 | 2.24 | Normal (1.79, 0.23) | (3) | 1.72 | 1.29 | 2.15 | Normal (1.72, 0.22) | (4) |
| Body weight (kg) | 70 | 52.50 | 87.50 | Normal (70, 8.93) | (5) | 65 | 48.75 | 81.25 | Normal (65, 8.29) | (4) |
| Discount rate | 0.03 | 0 | 0.08 | Uniform | (6) | 0.05 | 0 | 0.08 | Uniform | (6) |

^a^ The details of subsequent therapy were sourced from the OS analysis of AURA3 trial (2).

ITT, intention-to-treat; PFS, progression-free survival; PD, progressive disease; CNS, central nervous system metastases; HR, hazard ratio; SAEs, severe adverse events; AEs, adverse events; EGFR, epidermal growth factor receptor; VEGF, vascular endothelial growth factor; PD-1, programmed cell death protein 1; HER2, Human epidermal growth factor receptor-2; BSA, body surface area

**Supplementary Table 3.** Base-Case Cost Estimates and Utilities.

| **Parameter** | **United States** | | | | | **China** | | | | |
| --- | --- | --- | --- | --- | --- | --- | --- | --- | --- | --- |
|  | **Values** | **Range** | |  |  | **Values** | **Range** | |  |  |
|  |  | **Lower limit** | **Upper limit** | **Distribution (Parameters)** | **Reference** |  | **Lower limit** | **Upper limit** | **Distribution (Parameters)** | **Reference** |
| Drug Cost ($/mg) |  |  |  |  |  |  |  |  |  |  |
| Osimertinib ^a^ | 6.62 | 3.31 | 6.62 | Gamma (61.47, 9.28) | (7) | 0.36 | 0.11 | 0.36 | Gamma (31.86, 88.51) | (8) |
| Cisplatin | 0.19 | 0.14 | 0.24 | Gamma (61.47, 323.50) | (9) | 0.12 | 0.09 | 0.15 | Gamma (61.47, 512.21) | (8) |
| Pemetrexed | 7.29 | 5.47 | 9.11 | Gamma (61.47, 8.43) | (9) | 0.86 | 0.65 | 1.08 | Gamma (61.47, 71.47) | (8) |
| Afatinib | 8.78 | 6.59 | 10.98 | Gamma (61.47, 7.00) | (7) | 0.77 | 0.58 | 0.96 | Gamma (61.47, 79.83) | (8) |
| Docetaxel | 0.62 | 0.47 | 0.78 | Gamma (61.47, 99.14) | (9) | 2.33 | 1.75 | 2.91 | Gamma (61.47, 26.38) | (8) |
| Bevacizumab | 7.25 | 5.44 | 9.06 | Gamma (61.47, 8.48) | (9) | 2.32 | 1.74 | 2.90 | Gamma (61.47, 26.49) | (8) |
| Etoposide | 0.07 | 0.05 | 0.09 | Gamma (61.47, 878.08) | (9) | 0.01 | 0.008 | 0.013 | Gamma (61.47, 6146.56) | (8) |
| Crizotinib | 1.29 | 0.97 | 1.61 | Gamma (61.47, 47.65) | (7) | 0.14 | 0.11 | 0.18 | Gamma (61.47,439.04) | (8) |
| Vinorelbine | 1.07 | 0.80 | 1.34 | Gamma (61.47, 57.44) | (9) | 1.96 | 1.47 | 2.45 | Gamma (61.47, 31.36) | (8) |
| Selumetinib ^b^ | 7.91 | 5.93 | 9.89 | Gamma (61.47,7.77) | (7) | 7.91^b^ | 5.93 | 9.89 | Gamma (61.47,7.77) | (7) |
| Pembrolizumab | 51.62 | 38.72 | 64.53 | Gamma (61.47, 1.19) | (9) | 27.67 | 20.75 | 34.59 | Gamma (61.47, 2.22) | (10) |
| Necitumumab ^b^ | 5.74 | 4.31 | 7.18 | Gamma (61.47, 10.71) | (11) | 5.74^b^ | 4.31 | 7.18 | Gamma (61.47, 10.71) |  |
| Trastuzumab | 9.37 | 7.03 | 11.71 | Gamma (61.47, 6.56) | (9) | 1.93 | 1.45 | 2.41 | Gamma (61.47, 31.85) | (8) |
| Gemcitabine | 0.02 | 0.02 | 0.03 | Gamma (61.47, 3073.28) | (9) | 0.26 | 0.20 | 0.33 | Gamma (61.47, 236.41) | (8) |
| Radiotherapy cost ($/cycle) | 2265.26 | 1698.95 | 2831.58 | Gamma (61.47, 0.03) | (11) | 6605.91 | 4954.43 | 8257.39 | Gamma (61.47, 0.01) | (12) |
| Drug administration cost ($) |  |  |  |  |  |  |  |  |  |  |
| Chemotherapy infusion cost | 179.70 | 134.78 | 224.63 | Gamma (61.47, 0.34) | (1) | 20.63 | 15.47 | 25.79 | Gamma (61.47, 2.98) | (13) |
| Follow up cost per month | 474.78 | 356.09 | 593.48 | Gamma (61.47, 0.13) | (14) | 63.09 | 47.32 | 78.86 | Gamma (61.47, 0.97) | (15) |
| End-of-life cost | 9203.98 | 6902.99 | 11504.98 | Gamma (61.47, 0.01) | (16) | 1990.29 | 1492.72 | 2487.86 | Gamma (61.47, 0.03) | (15) |
| Best supportive care cost per month | 2254.19 | 1690.64 | 2817.74 | Gamma (61.47, 0.03) | (17) | 78.59 | 58.94 | 98.24 | Gamma (61.47, 0.78) | (15) |
| EGFR mutation testing | 1047.80 | 785.85 | 1309.75 | Gamma (61.47, 0.06) | (18) | 1500 | 1125.00 | 1875.00 | Gamma (61.47, 0.04) | Local charge |
| Adverse event costs ($/per event) |  |  |  |  |  |  |  |  |  |  |
| Neutropenia | 19251.90 | 14438.93 | 24064.88 | Gamma (61.47, 0.003) | (19) | 2725.98 | 2044.49 | 3407.48 | Gamma (61.47,0.02) | (20) |
| Anemia | 22702.02 | 17026.52 | 28377.53 | Gamma (61.47, 0.003) | (19) | 6907.82 | 5180.87 | 8634.78 | Gamma (61.47, 0.01) | (21) |
| Thrombocytopenia | 25433.88 | 19075.41 | 31792.35 | Gamma (61.47, 0.002) | (19) | 6537.25 | 4902.94 | 8171.56 | Gamma (61.47, 0.01) | (22) |
| Utilities |  |  |  |  |  |  |  |  |  |  |
| PFS (Osimertinib group) | 0.82 | 0.83 | 0.84 | Beta (4649.57, 1020.64) | (23) | 0.79 | 0.74 | 0.83 | Beta (249.39, 67.50) | (24) |
| PFS (Platinum-pemetrexed group) | 0.78 | 0.75 | 0.81 | Beta (571.32, 161.14) | (23) | 0.84 | 0.81 | 0.98 | Beta (62.65, 12.11) | (24) |
| PD | 0.73 | 0.71 | 0.75 | Beta (1381.85, 511.10) | (23) | 0.70 | 0.66 | 0.75 | Beta (254.83, 107.66) | (24) |
| SAEs disutility |  |  |  |  |  |  |  |  |  |  |
| Neutropenia | 0.20 | 0.15 | 0.25 | Beta (49.17, 196.69) | (25) | 0.20 | 0.15 | 0.25 | Beta (49.17, 196.69) | (25) |
| Anemia | 0.09 | 0.07 | 0.11 | Beta (55.93, 565.55) | (26) | 0.09 | 0.07 | 0.11 | Beta (55.93, 565.55) | (26) |
| Thrombocytopenia | 0.20 | 0.15 | 0.25 | Beta (49.17, 196.69) | (12) | 0.20 | 0.15 | 0.25 | Beta (49.17, 196.69) | (12) |

^a^ In the United States, the price of osimertinib would be discounted at 17% to account for contract pricing (27). In China, the price of osimertinib would be discounted by 30% to account for the out-of-pocket ratio after medical insurance reimbursement (8).

^b^ The price of selumetinib and necitumumab in China was assumed consistent with that in the United States due to selumetinib and necitumumab are not available.

PFS, progression-free survival; PD, progressive disease; SAEs, severe adverse events; EGFR, epidermal growth factor receptor; VEGF, vascular endothelial growth factor; PD-1, programmed cell death protein 1.

**Supplementary Table 4.** R statistics code: output for the common parametric survival distributions.

| **Parametric model** | **Survivor function** | **R statistics code** |
| --- | --- | --- |
| Exponential | $S(t)=\exp(-\lambda t)$ | **λ**<-exp(-intercept) |
| Weibull | $S(t)=\exp(-\lambda t^{\gamma})$ | **λ** <- 1/ (exp(intercept))^ (1/exp(log_scale))  **γ** <- 1/exp(log_scale) |
| Log-logistic | $S(t)=\frac{1}{1+\lambda t^{\gamma}}$ | **λ**<-exp(-(intercept)/scale)  **γ** <- 1/exp(log_scale) |
| Lognormal | $S(t)=1-\varphi((Lnt-\mu)/\sigma)$ | u<-intercept  σ<-scale |

$S(t)$, probability of surviving beyond time t; $\lambda$, scale parameter; $\gamma$, shape parameter; $\mu$, mean value; $\sigma$, the standard deviation

**Supplementary Table 5.** Summary of statistical goodness-of-fit of Kaplan-Meier curve in AURA3 trial.

|  | **Exponential** | **Weibull** | **Log-logistic** | **Lognormal** |
| --- | --- | --- | --- | --- |
| **ITT population** |  |  |  |  |
| PFS curve of osimertinib |  |  |  |  |
| AIC | 1122.456 | 1103.579 | 1102.244 | 1103.626 |
| BIC | 1126.083 | 1110.834 | 1109.500 | 1110.882 |
| PFS curve of platinum-pemetrexed |  |  |  |  |
| AIC | 714.335 | 701.005 | 705.450 | 708.878 |
| BIC | 717.318 | 706.972 | 711.417 | 714.845 |
| OS curve of osimertinib |  |  |  |  |
| AIC | 1867.653 | 1838.605 | 1836.901 | 1848.494 |
| BIC | 1871.281 | 1845.860 | 1844.156 | 1855.749 |
| OS curve of platinum-pemetrexed |  |  |  |  |
| AIC | 917.098 | 916.579 | 918.222 | 926.400 |
| BIC | 920.047 | 922.476 | 924.120 | 932.297 |
| **Patients with CNS metastases** |  |  |  |  |
| PFS curve of osimertinib |  |  |  |  |
| AIC | 374.073 | 367.765 | 364.519 | 363.985 |
| BIC | 376.595 | 372.808 | 369.563 | 369.029 |
| PFS curve of platinum-pemetrexed |  |  |  |  |
| AIC | 243.847 | 234.555 | 241.750 | 244.637 |
| BIC | 245.718 | 238.298 | 245.492 | 248.379 |

ITT, intention-to-treat; OS, overall survival; PFS, progression-free survival; AIC, Akaike’s information criterion; BIC, Bayesian information criterion; CNS, Central nervous system

The visual fits and statistical fits of four parametric survival models including exponential, Weibull, log-logistic, and lognormal are presented in Supplementary Table 3.

In the ITT population, as for the platinum-pemetrexed PFS curve, Weibull distribution had the lowest AIC and BIC. Therefore, Weibull distribution may be appropriate for PFS of platinum-pemetrexed. As for the osimertinib PFS curve, log-logistic distribution had the lowest AIC and BIC. Therefore, log-logistic distribution may be appropriate for the OS of osimertinib. As for the platinum-pemetrexed OS curve, Weibull and exponential distribution had the lowest AIC and BIC, respectively. However, BIC is more credible with a bigger penalty, AIC is preferred for large samples. Therefore, the Weibull distribution was likely to be the most reasonable parametric model for OS of platinum-pemetrexed. As for the osimertinib OS curve, log-logistic distribution had the lowest AIC and BIC for OS of Osimertinib. However, the log-logistic models typically have long tails due to a reducing hazard as time increases after a certain point, which would likely overestimate OS in the long term based on clinical experts’ opinions. Therefore, Weibull distribution may be more appropriate for OS of osimertinib.

In patients with CNS metastases, as for the platinum-pemetrexed PFS curve, Weibull distribution had the lowest AIC and BIC. Therefore, Weibull distribution may be appropriate for PFS of platinum-pemetrexed. As for the osimertinib PFS curve, lognormal distribution had the lowest AIC and BIC. However, when the lognormal distribution is selected, the PFS curve of the CNS population is always higher than that of the ITT population during 4.5 years of follow-up from enrollment, which meant the number of patients keeping in PFS in patients with CNS metastases was higher than that of in ITT population. This is not consistent with clinical practice. Considering AIC, BIC, and clinical rationality, log-logistic distribution was considered to be a more appropriate parametric model for the osimertinib PFS curve in patients with CNS metastases.

**Supplementary Table 6.** CHEERS checklist.

| Section/item | Item No | Recommendation | Reported? |
| --- | --- | --- | --- |
| Title and abstract | | | |
| Title | 1 | Identify the study as an economic evaluation or use more specific terms such as “cost-effectiveness analysis”, and describe the interventions compared. | Yes |
| Abstract | 2 | Provide a structured summary of objectives, perspective, setting, methods (including study design and inputs), results (including base case and uncertainty analyses), and conclusions. | Yes |
| Introduction | | | |
| Background and objectives | 3 | Provide an explicit statement of the broader context for the study. Present the study question and its relevance for health policy or practice decisions. | Yes |
| Methods | | | |
| Target population and subgroups | 4 | Describe characteristics of the base case population and subgroups analyzed, including why they were chosen. | Yes |
| Setting and location | 5 | State relevant aspects of the system(s) in which the decision(s) need(s) to be made | Yes |
| Study perspective | 6 | Describe the perspective of the study and relate this to the costs being evaluated. | Yes |
| Comparators | 7 | Describe the interventions or strategies being compared and state why they were chosen. | Yes |
| Time horizon | 8 | State the time horizon(s) over which costs and consequences are being evaluated and say why appropriate. | Yes |
| Discount rate | 9 | Report the choice of discount rate(s) used for costs and outcomes and say why appropriate. | Yes |
| Choice of health outcomes | 10 | Describe what outcomes were used as the measure(s) of benefit in the evaluation and their relevance for the type of analysis performed. | Yes |
| Measurement of effectiveness | 11a | Single study-based estimates: Describe fully the design features of the single effectiveness study and why the single study was a sufficient source of clinical effectiveness data. | Yes |
|  | 11b | Synthesis-based estimates: Describe fully the methods used for identification of included studies and synthesis of clinical effectiveness data. | N/A |
| Measurement and valuation of preference based outcomes | 12 | If applicable, describe the population and methods used to elicit preferences for outcomes. | N/A |
| Estimating resources and costs | 13a | Single study-based economic evaluation: Describe approaches used to estimate resource use associated with the alternative interventions. Describe primary or secondary research methods for valuing each resource item in terms of its unit cost. Describe any adjustments made to approximate to opportunity costs. | N/A |
|  | 13b | Model-based economic evaluation: Describe approaches and data sources used to estimate resource use associated with model health states. Describe primary or secondary research methods for valuing each resource item in terms of its unit cost. Describe any adjustments made to approximate to opportunity costs. | Yes |
| Currency, price date, and conversion | 14 | Report the dates of the estimated resource quantities and unit costs. Describe methods for adjusting estimated unit costs to the year of reported costs if necessary. Describe methods for converting costs into a common currency base and the exchange rate. | Yes |
| Choice of model | 15 | Describe and give reasons for the specific type of decision-analytical model used. Providing a figure to show model structure is strongly recommended. | Yes |
| Assumptions | 16 | Describe all structural or other assumptions underpinning the decision-analytical model. | Yes |
| Analytical methods | 17 | Describe all analytical methods supporting the evaluation. This could include methods for dealing with skewed, missing, or censored data; extrapolation methods;  Yes  methods for pooling data; approaches to validate or make adjustments (such as half cycle corrections) to a model; and methods for handling population heterogeneity and uncertainty. | Yes |
| Results | | | |
| Study parameters | 18 | Report the values, ranges, references, and, if used, probability distributions for all parameters. Report reasons or sources for distributions used to represent uncertainty where appropriate. Providing a table to show the input values is strongly recommended. | Yes |
| Incremental costs and outcomes | 19 | For each intervention, report mean values for the main categories of estimated costs and outcomes of interest, as well as mean differences between the comparator groups. If applicable, report incremental cost-effectiveness ratios. | Yes |
| Characterizing uncertainty | 20a | Single study-based economic evaluation: Describe the effects of sampling uncertainty for the estimated incremental cost and incremental effectiveness parameters, together with the impact of methodological assumptions (such as discount rate, study perspective). | N/A |
|  | 20b | Model-based economic evaluation: Describe the effects on the results of uncertainty for all input parameters, and uncertainty related to the structure of the model and assumptions. | Yes |
| Characterizing heterogeneity | 21 | If applicable, report differences in costs, outcomes, or cost-effectiveness that can be explained by variations between subgroups of patients with different baseline characteristics or other observed variability in effects that are not reducible by more information. | Yes |
| Discussion | | | |
| Study findings, limitations, generalizability, and current knowledge | 22 | Summarize key study findings and describe how they support the conclusions reached. Discuss limitations and the generalizability of the findings and how the findings fit with current knowledge. | Yes |
| Other | | | |
| Source of funding | 23 | Describe how the study was funded and the role of the funder in the identification, design, conduct, and reporting of the analysis. Describe other non-monetary sources of support. | Yes |
| Conflicts of interest | 24 | Describe any potential for conflict of interest of study contributors in accordance with journal policy. In the absence of a journal policy, we recommend authors comply with International Committee of Medical Journal Editors recommendations. | Yes |

**REFERENCES**

1. Mok TS, Wu YL, Ahn MJ, Garassino MC, Kim HR, Ramalingam SS, et al. Osimertinib or Platinum-Pemetrexed in EGFR T790M-Positive Lung Cancer. *N Engl J Med* (2017) 376(7): 629-640. doi: 10.1056/NEJMoa1612674.

2. Papadimitrakopoulou VA, Mok TS, Han JY, Ahn MJ, Delmonte A, Ramalingam SS, et al. Osimertinib versus platinum-pemetrexed for patients with EGFR T790M advanced NSCLC and progression on a prior EGFR-tyrosine kinase inhibitor: AURA3 overall survival analysis. *Ann Oncol* (2020) 31(11): 1536-1544. doi: 10.1016/j.annonc.2020.08.2100.

3. Goulart B, and Ramsey S. A trial-based assessment of the cost-utility of bevacizumab and chemotherapy versus chemotherapy alone for advanced non-small cell lung cancer. *Value Health* (2011) 14(6): 836-845. doi: 10.1016/j.jval.2011.04.004.

4. Wu B, Chen HF, Shen JF, and Ye M. Cost-effectiveness of adding rhendostatin to first-line chemotherapy in patients with advanced non-small-cell lung cancer in China. *Clin Ther* (2011) 33(10): 1446-1455. doi: 10.1016/j.clinthera.2011.09.016.

5. Kohn CG, Zeichner SB, Chen Q, Montero AJ, Goldstein DA, and Flowers CR. Cost-Effectiveness of Immune Checkpoint Inhibition in BRAF Wild-Type Advanced Melanoma. *J Clin Oncol* (2017) 35(11): 1194-1202. doi: 10.1200/JCO.2016.69.6336.

6. Neumann PJ, Cohen JT, and Weinstein MC. Updating cost-effectiveness--the curious resilience of the $50,000-per-QALY threshold. *N Engl J Med* (2014) 371(9): 796-797. doi: 10.1056/NEJMp1405158.

7. National Bureau of Statistics of China, Health care and personal articles of consumer price indices. Available at: <http://data.stats.gov.cn/search.htm?s=CPI>, 2021 (Accessed March 9 2021).

8. Centers for Medicare & Medicaid Services, Hospital Outpatient PPS: Addendum A and Addendum B updates. Available at: <https://www.cms.gov/Medicare/Medicare-Fee-for-Service-Payment/HospitalOutpatientPPS/Addendum-A-and-Addendum-B-Updates>, 2021 (Accessed March 9 2021).

9. Cao W, Chen HD, Yu YW, Li N, and Chen WQ. Changing profiles of cancer burden worldwide and in China: a secondary analysis of the global cancer statistics 2020. *Chin Med J (Engl)* (2021) 134(7): 783-791. doi: 10.1097/CM9.0000000000001474.

10. Centers for Medicare & Medicaid Services, 2021 Physician Fee Schedule Search. Available at: <https://www.cms.gov/medicare/physician-fee-schedule/search?Y=0&T=4&HT=0&CT=3&H1=96413&M=5>, 2021 (Accessed March 9 2021).

11. Yoon SM, Shaikh T, and Hallman M. Therapeutic management options for stage III non-small cell lung cancer. *World J Clin Oncol* (2017) 8(1): 1-20. doi: 10.5306/wjco.v8.i1.1.

12. Guan HJ, Liu G, Xie F, Sheng Y, and Shi L. Cost-effectiveness of Osimertinib as a Second-line Treatment in Patients With EGFR-mutated Advanced Non-Small Cell Lung Cancer in China. *Clin. Ther.* (2019) 41(11): 2308-2320 e2311. doi: 10.1016/j.clinthera.2019.09.008.

13. Shi Y, Wan XM, Tan CQ, Li JH, and Peng LB. Model-Based Cost-Effectiveness Analysis of Panitumumab Plus FOLFIRI for the Second-Line Treatment of Patients with Wild-Type Ras Metastatic Colorectal Cancer. *Adv Ther* (2020) 37(2): 847-859. doi: 10.1007/s12325-019-01214-y.

14. Klein R, Wielage R, Muehlenbein C, Liepa AM, Babineaux S, Lawson A, et al. Cost-effectiveness of pemetrexed as first-line maintenance therapy for advanced nonsquamous non-small cell lung cancer. *J Thorac Oncol* (2010) 5(8): 1263-1272. doi: 10.1097/JTO.0b013e3181e15d16.

15. Li HC, Lai L, and Wu B. Cost Effectiveness of Ceritinib and Alectinib Versus Crizotinib in First-Line Anaplastic Lymphoma Kinase-Positive Advanced Non-small-cell Lung Cancer. *Clin Drug Investig* (2020) 40(2): 183-189. doi: 10.1007/s40261-019-00880-8.

16. Aguiar PN, Jr., Haaland B, Park W, San Tan P, Del Giglio A, and de Lima Lopes G, Jr. Cost-effectiveness of Osimertinib in the First-Line Treatment of Patients With EGFR-Mutated Advanced Non-Small Cell Lung Cancer. *JAMA Oncol* (2018) 4(8): 1080-1084. doi: 10.1001/jamaoncol.2018.1395.

17. Criss SD, Mooradian MJ, Sheehan DF, Zubiri L, Lumish MA, Gainor JF, et al. Cost-effectiveness and Budgetary Consequence Analysis of Durvalumab Consolidation Therapy vs No Consolidation Therapy After Chemoradiotherapy in Stage III Non-Small Cell Lung Cancer in the Context of the US Health Care System. *JAMA Oncol* (2019) 5(3): 358-365. doi: 10.1001/jamaoncol.2018.5449.

18. Sands J, Li Q, and Hornberger J. Urine circulating-tumor DNA (ctDNA) detection of acquired EGFR T790M mutation in non-small-cell lung cancer: An outcomes and total cost-of-care analysis. *Lung Cancer* (2017) 110: 19-25. doi: 10.1016/j.lungcan.2017.05.014.

19. Wong W, Yim YM, Kim A, Cloutier M, Gauthier-Loiselle M, Gagnon-Sanschagrin P, et al. Assessment of costs associated with adverse events in patients with cancer. *PLoS One* (2018) 13(4): e0196007. doi: 10.1371/journal.pone.0196007.

20. Liu GQ, and Kang S. Cost-effectiveness of adding durvalumab to first-line chemotherapy for extensive-stage small-cell lung cancer in China. *Expert Rev Pharmacoecon Outcomes Res* (2021): 1-7. doi: 10.1080/14737167.2021.1888717.

21. Gu X, Zhang Q, Chu YB, Zhao YY, Zhang YJ, Kuo D, et al. Cost-effectiveness of afatinib, gefitinib, erlotinib and pemetrexed-based chemotherapy as first-line treatments for advanced non-small cell lung cancer in China. *Lung Cancer* (2019) 127: 84-89. doi: 10.1016/j.lungcan.2018.11.029.

22. Wan N, Zhang TT, Hua SH, Lu ZL, Ji B, Li LX, et al. Cost-effectiveness analysis of pembrolizumab plus chemotherapy with PD-L1 test for the first-line treatment of NSCLC. *Cancer Med* (2020) 9(5): 1683-1693. doi: 10.1002/cam4.2793.

23. Labbe C, Leung Y, Silva Lemes JG, Stewart E, Brown C, Cosio AP, et al. Real-World EQ5D Health Utility Scores for Patients With Metastatic Lung Cancer by Molecular Alteration and Response to Therapy. *Clin Lung Cancer* (2017) 18(4): 388-395 e384. doi: 10.1016/j.cllc.2016.12.015.

24. Shen Y, Wu B, Wang X, and Zhu J. Health state utilities in patients with advanced non-small-cell lung cancer in China. *J Comp Eff Res* (2018) 7(5): 443-452. doi: 10.2217/cer-2017-0069.

25. Nafees B, Lloyd AJ, Dewilde S, Rajan N, and Lorenzo M. Health state utilities in non-small cell lung cancer: An international study. *Asia. Pac. J. Clin. Oncol.* (2017) 13(5): e195-e203. doi: 10.1111/ajco.12477.

26. Beusterien KM, Davies J, Leach M, Meiklejohn D, Grinspan JL, O'Toole A, et al. Population preference values for treatment outcomes in chronic lymphocytic leukaemia: a cross-sectional utility study. *Health Qual Life Outcomes* (2010) 8: 50. doi: 10.1186/1477-7525-8-50.

27. Hornberger J, Hirsch FR, Li Q, and Page RD. Outcome and economic implications of proteomic test-guided second- or third-line treatment for advanced non-small cell lung cancer: extended analysis of the PROSE trial. *Lung Cancer* (2015) 88(2): 223-230. doi: 10.1016/j.lungcan.2015.03.006.
